# Periodic blinking manipulation of magnetic Janus particles with a tunable electromagnetic field for rapid sensing of extracellular vesicles

**DOI:** 10.3389/fbioe.2025.1565479

**Published:** 2025-04-25

**Authors:** Han-Sheng Chuang, Thi Thanh Huong Pham, Yu-Hsuan Chou, Chi-Ying F. Huang, Ting-Yuan Tu, Tai-Hua Yang, Jhih-Cheng Wang

**Affiliations:** ^1^ Department of Biomedical Engineering, National Cheng Kung University, Tainan, Taiwan; ^2^ Medical Device Innovation Center, National Cheng Kung University, Tainan, Taiwan; ^3^ Institute of Biopharmaceutical Sciences, National Yang Ming Chiao Tung University, Taipei, Taiwan; ^4^ Department of Orthopedic Surgery, National Cheng Kung University Hospital, Tainan, Taiwan; ^5^ Department of Urology, Chimei Medical Center, Tainan, Taiwan; ^6^ Department of Electrical Engineering, Southern Taiwan University of Science and Technology, Tainan, Taiwan; ^7^ School of Medicine, College of Medicine, National Sun Yat-sen University, Kaohsiung, Taiwan

**Keywords:** magnetic Janus particles, extracellular vesicles, biosensors, immunoassays, tunable electromagnet, cutoff frequency, magnetic field

## Abstract

Detecting small biological molecules is challenging due to their tiny size, vulnerability, and low concentrations in samples. Bead-based biosensors are frequently used as probes but require tedious processing or expensive instruments. By combining magnetic Janus particles (MJPs) and an electromagnetic device, we successfully built an active diagnostic tool for the rapid sensing of small extracellular vesicles (sEVs). We observed that the system can be altered according to particle size, distance between MJPs and the electromagnet, fluid viscosity, and magnetic field strength. By modulating the driving frequency from low (3 Hz) to high (22 Hz), the MJPs gradually lose their synchrony with the external magnetic field after exceeding a certain threshold termed cutoff frequency. The novel sEVs sensing MJP system was characterized through both theoretical and experimental methods, showing reliable performance in identifying the cancer cell OECM-1-derived sEVs using the CD63 surface marker. A decent sEV concentration of 2.9 × 10^9^ particles mL^−1^ was reached and a high specificity was also observed. This approach opens a door for the realization of disease screening, such as cancer, using intact exosomes from body fluids without sophisticated processing. These findings provide insight into the future use of MJPs as point-of-care testing tools for liquid biopsy.

## 1 Introduction

Small biological molecules, such as cytokines, miRNAs, and extracellular vesicles (EVs), have been widely reported as potential biomarkers for various diseases, such as cancer (Mathew et al., 2020; Kartikasari et al., 2021; Metcalf, 2024) and neurological disorders (Espíndola et al., 2021; Raghav et al., 2022; Musso et al., 2023). Exosomes, also known as small EVs (sEVs), have attracted considerable interest because of their high homology with host cells (Zhang et al., 2019; Qiao et al., 2020). They are EVs originating from the cell’s endosomal system. In the biogenesis of exosomes, specific proteins, lipids, and nucleic acids are first packaged in multivesicular bodies (MVBs). After the MVBs fuse with the plasma membrane of a cell, the contents encapsulated in multiple small vesicles are released into the extracellular space and then become sEVs. Typical sEVs range in size from 30 to 150 nm and carry specific membrane markers, such as CD63, CD81, and CD9 (Lu et al., 2021; Fan et al., 2023). Many sEVs exist in body fluids, including serum, urine, saliva, and tears, which make them easy to collect and analyze. These characteristics make sEVs ideal targets for diagnosis or treatments. The common practices of current exosome diagnosis are based on tedious processing, which requires lysing sEVs to release their loads and isolating target contents through multiple washing and filtration steps. Various diagnostic tools, such as liquid chromatography–mass spectrometry (Schey et al., 2015), flow cytometry (Morales-Kastresana and Jones, 2017), enzyme-linked immunosorbent assay (Lee J. et al., 2020; Logozzi et al., 2020), next-generation sequencing (Yagi et al., 2017; Elkommos-Zakhary et al., 2022), and qPCR (Bellingham et al., 2017; Lee H. et al., 2020), are commonly used in identifying potential sEVs for disease diagnoses. However, existing approaches are time-consuming, labor intensive, costly, and incapable of point-of-care testing (POCT). Alternatively, emerging techniques capable of detecting small biological molecules have been proposed to improve exosome-based disease diagnosis. Doldán et al. (2016) developed an electrochemical sandwich immunosensor for determining breast cancer cell–derived exosomes; in their electrochemical biosensor, exosomes were sandwiched by two types of CD9 antibodies: one that immobilized the exosomes on electrodes and the other that served as a secondary antibody conjugate with horseradish peroxidase (HRP); amperometric signals were measured on the basis of the redox reaction of TMB catalyzed by HRP as a reporter; with this approach, they achieved a limit of detection (LoD) of 2 × 10^2^ exosomes mL^−1^ in a sample volume of as low as 1.5 μL and a dynamic measurement range spanning four orders of magnitude; their work provides a potential aid for the determination of EVs in clinical samples and potentially offsets the need for expensive equipment and laborious purification. Zhang et al. (2021) demonstrated electrochemical micro-aptasensors for EpCAM exosome detection based on hybridization chain reaction amplification. A wide range of exosome concentrations from 2.5 × 10^3^ to 1 × 10^7^ exosomes mL^−1^ with an LoD of approximately 5 × 10^2^ exosomes/mL was eventually achieved; this approach successfully detected early- and late-stage lung cancer by using exosomes in serum samples. Kwizera et al. (2018) used surface-enhanced Raman scattering gold nanorods (SERS-AuNRs) to detect breast cancer–derived exosomes; the device was composed of a gold-coated glass slide and plastic array templates, where exosomes were captured in wells with anti-CD63; the SERS-AuNRs were attached to exosomal lipid membranes to enhance signals; their findings showed that EpCAM, CD44, and HER2 are biomarkers for distinguishing breast cancer exosomes from normal cell-derived ones; an LoD of 2 × 10^6^ exosomes mL^−1^ and analysis of over 80 samples on a single device within 2 h were achieved. Zhang et al. (2023) presented a label- and antibody-free impedimetric biosensor based on molecularly imprinting technology for detecting exosomes derived from non-small-cell lung cancer cells (A549); a selective adsorption membrane for A549 exosomes was created using anchored template exosomes on a glassy carbon electrode (GCE); the concentration of captured exosomes was monitored according to the impedance change of the GCE; their approach eventually achieved an LoD of 2.03 × 10^3^ exosomes mL^−1^, excellent accuracy and precision, recovery ratio of 100.76%, and relative standard deviation of 1.86%. By combining spiky-shaped aptamer-magnetic beads (Au@Fe_3_O_4_/Apt) with an electrochemical platform, Pan et al. (2022) developed a novel biosensor for the detection of cancer-derived exosomes. The spiky nanobeads facilitated exosome enrichment and signal amplification, thereby significantly enhancing the sensitivity of the biosensor. The optimal LoD eventually reached 8 × 10^4^ exosomes mL^−1^. Another exosome detection method based on aptamer-modified magnetic nanoparticles was reported by Yu et al. (2019). Initially, a Cy3 label was hybridized with the aptamer to express fluorescence. However, the Cy3 dye would be shed in the presence of exosomes due to the high affinity between the aptamer and the transmembrane protein CD63. As a result, an LOD of 1 × 10^8^ exosomes mL^−1^ was achieved. Despite these impressive results, most of these technologies are still under development and need to be validated with clinical trials.

Alternatively, we present an active magnetic Janus particle (MJP) system manipulated by an external electromagnet to detect potential disease biomarkers. Our prior work based on plain Janus-particle biosensors showcased the feasibility of detecting different biological targets, such as bacteria (Chung et al., 2016, 2017; Wang et al., 2018; Yang et al., 2020), proteins (Chuang et al., 2018; Cheng and Chuang, 2019), and nucleic acids (Wang et al., 2020; Das et al., 2022a; Das et al., 2022b; Das et al., 2024) on the basis of Brownian motion. With active control, MJPs can function in broad environments and have improved signal-to-noise ratios. Current MJPs are fabricated by coating three thin films, namely, silver (5 nm), nickel (15 nm), and gold (5 nm), on the half of a substrate containing 1-μm polystyrene (PS) fluorescent particles. The nickel layer enables MJPs to respond to any magnetic stimulus. When MJPs rotate, partially covered fluorescent particles exhibit a blinking effect that can be quantified in terms of frequency. Functionalized antibodies, such as anti-CD63 IgG, on the gold surfaces of MJPs allow for the capture of target sEVs through corresponding membrane markers, including CD63. sEVs attached to MJPs increase the effective volume of particles, inhibiting their capability to follow an external magnetic field. Kopelman and his colleagues previously proposed a similar concept, termed asynchronous magnetic bead rotation (AMBR), to investigate small changes in viscosity after DNA amplification (Li et al., 2014) and bacterial growth (Sinn et al., 2011). Their findings showed that a magnetic bead’s critical slipping rate is proportional to fluid viscosity and bead volume but inversely proportional to magnetic field strength, indicating that AMBRs can be used in measuring multiple physical properties. Our proposed setup can be further miniaturized and simplified owing to the compact magnetic device after the rotating magnet is replaced with a nonmoving electromagnet. When magnetic polarity is changed (S and N poles), MJPs rotate back and forth in alignment with the magnetic field. However, MJPs tend to deviate from the field at a high driving frequency, dubbed cutoff frequency, which is a function of fluid viscosity, volume of the bead, and magnetic field strength. By controlling any two parameters, a third unknown parameter can be easily obtained. Theoretical predictions and experimental measurements related to these three parameters have been investigated, and optimal conditions have been determined. Theoretical predictions and experimental measurements showed similar trends. A minimum detected concentration of 2.9 × 10^9^ particles mL^−1^ was reached for sEVs. The promising MJP system offers insight into revolutionized POCT as a novel approach targeting sEVs that can be performed at any time as a preventive measure. Realization of this approach is quite flexible since only MJPs and an electromagnet need to be deployed. Combined with standard check-ups, such as imaging tests (X-ray, ultrasound, CT, MRI scans), the MJP system, when modified with appropriate disease markers, can provide deeper insights for high-risk patients, enhancing early intervention and personalized care.

## 2 Materials and methods

### 2.1 Fabrication and characterizations of MJPs

MJPs were fabricated by following our previously developed protocol (Chen and Chuang, 2020). To synthesize these MJPs, half of each 1-μm fluorescent PS particles (F13083, ThermoFisher, MA, United States) was coated with metallic layers. This asymmetric coating caused the MJPs to exhibit blinking fluorescence when suspended in a medium. In contrast to our prior plain Janus particles, nickel was incorporated into the metallic coating to enable responsiveness to external magnetic fields. The metallic layers were sequentially deposited on the PS particles in the following order (innermost to outermost): 5-nm silver, 15-nm nickel, and 5-nm gold. The innermost silver coating served as an adhesion between the particle surface and the above metals. The middle nickel coating functioned as a magnetic source in response to an external magnetic field. The outermost gold coating was used to facilitate subsequent functionalization.

For the coating of the fluorescent particles, 1-μm fluorescent PS particles were first suspended in a 95% ethanol solution and then air-dried on a hydrophobic glass slide, which was coated with 1% Cytop (CTL-109AE, AGC Chemicals, Japan; diluted in solvent CT-SOLV100E). A monolayer of the PS particles was evenly spread over the surface of the glass slide after rapid evaporation. The glass slide was then sent to an evaporator for sequential coatings with silver (5 nm), nickel (15 nm), and gold (5 nm). The inner silver coating served as an adhesion layer, the middle nickel coating was used for magnetic attraction, and the outer gold coating was used to facilitate antibody conjugation. Subsequently, the coated glass slide was immersed in a Petri dish filled with phosphate buffered saline (PBS) buffer (IB3011, OmicsBio, Taiwan). MJPs were collected by placing the whole Petri dish in a water bath, sonicated for 3 h, and transferred to a water solution containing 1% (v/v) Tween 20 (P5927, Sigma-Aldrich, MO, United States). For purification, two-step filtration was conducted using membranes with 5 and 3 μm pores. After intensive purification, most gold debris and impurities were effectively removed (Supplementary Figure S1). The final suspension was stored at 4°C for later surface modifications.

Purified MJPs were examined with SEM (Helios G4, ThermoFisher Scientific, MA, United States). In the SEM images of secondary electrons (Supplementary Figure S2A) and backscattered electrons (Supplementary Figure S2B), the MJPs were half coated as intended. Element analysis was performed on the MJPs with an energy dispersive X-ray spectrometer (Supplementary Figures S2C,D). The composition of the coatings, including silver, nickel, and gold, were basically consistent with our design. The results confirmed that the MJPs can serve as biosensors for detecting small molecules.

### 2.2 Preparation of isolated sEVs

The oral cancer cell line OECM-1 was provided by Professor Wen-Tai Chiu at the Department of Biomedical Engineering of National Cheng Kung University, Taiwan. The cells were cultured in 15 cm cell culture dishes with RPMI 1640 medium (Simply Biologics, GeneDireX Inc., United States) supplemented with 10% fetal bovine serum (FBS; 16000044, ThermoFisher, MA, United States). Once the cells reached approximately 80% confluence, they were washed thrice with 1× PBS. The culture was then continued in fresh RPMI 1640 medium without FBS for at least 24 h. Then, the conditioned medium was collected for subsequent exosome isolation through ultracentrifugation (Supplementary Figure S3).

One hundred mL of the collected conditioned medium mentioned above was preprocessed by centrifugation at 500 × g for 10 min to remove cells and large cell debris. Then, filtration with a 0.22 µm filter (Simply Biologics, GeneDireX Inc., United States) was performed. The filtrate was transferred to sterilized polycarbonate ultracentrifuge tubes and set to spin in the precooled rotor of an ultracentrifuge (Beckman Optima XPN-90) at 100,000 × g for 4 h at 4°C. The initial sEV pellet was washed and resuspended in 30 mL of ice-cold sterile 1 × PBS before the second round of centrifugation at 100,000 × g for 2 h at 4°C. Final sEV isolates were harvested into a 1.5 mL microcentrifuge tube and kept in −80°C for preservation. The size distribution and concentration of the sEVs were assessed in triplicate using nanoparticle tracking analysis (NTA; NanoSight LM10-HS, Malvern Instruments, United Kingdom).

### 2.3 Functionalization of MJP immunocomplexes

The CD63 antibody (GTX41878, Genetex, Taiwan) was functionalized on the gold-coated half of the 1-μm MJP with a gold conjugation kit (40 nm, 20 OD, Abcam, United Kingdom). Stock MJPs were dispersed evenly by ultrasonication for 20 s and thoroughly vortex before the experiment. First, 2.4 μL of stock antibody (0.5 μg mL^−1^) was diluted to 0.1 μg mL^−1^ with 9.6 μL of gold antibody diluent to yield a total volume of 12 μL. Next, 42 μL of a gold reaction buffer was added, and 45 μL of the mixture was subsequently incubated with 20 μL of MJPs on a shaker at 800 rpm and room temperature for 15 min. To stop the reaction, 5 μL of a gold quencher buffer was gently mixed with the anti-CD63-functionalized MJP solution. After overnight storage at 4°C, the unbound antibodies were washed away thrice with 1 × PBS and centrifuged for 6 min at 13,500 rpm and room temperature.

For the detection of sEVs, stock sEV isolates were prepared using the method described in Section 2.2. Ultimately, three concentrations of sEV isolates were prepared with 1 × PBS: undiluted (original concentration), tenfold dilution, and 100-fold dilution. PBS and 0.1% bovine serum albumin (BSA; A7030, Sigma-Aldrich, MO, United States) served as the control references for validation and specificity of binding, respectively. To form immunocomplexes between functionalized MJPs and sEVs, 20 μL of the conjugated MJP solution was added to 10 μL of each sample. The mixture was incubated on a shaker (800 rpm, RT) for 1 h. Finally, a washing step with 1 × PBS was repeated thrice to eliminate uncaptured vesicles.

To verify the presence of the MJP-sEV immunocomplex formation, a mock colocalization of 1-μm MJP-sEV-200-nm green fluorescent PS bead immunocomplex was employed. To this end, anti-CD81 IgG (GTX31381, Genetex, Taiwan) was first conjugated to the 200-nm fluorescent PS beads (FluoSpheres carboxylate-modified microspheres, F8811, ThermoFisher, MA, United States) using EDC/NHS chemistry. Subsequently, 20 μL of the anti-CD81 IgG-conjugated green PS particle solution was added to 10 μL of the MJP-sEV mixture. Incubation and washing steps identical to those used in the previous MJP-sEV processing were then performed to form the final MJP-sEV-fluorescent PS bead immunocomplex.

### 2.4 Mechanism and operating strategy of the MJP biosensing system

**FIGURE 1:**
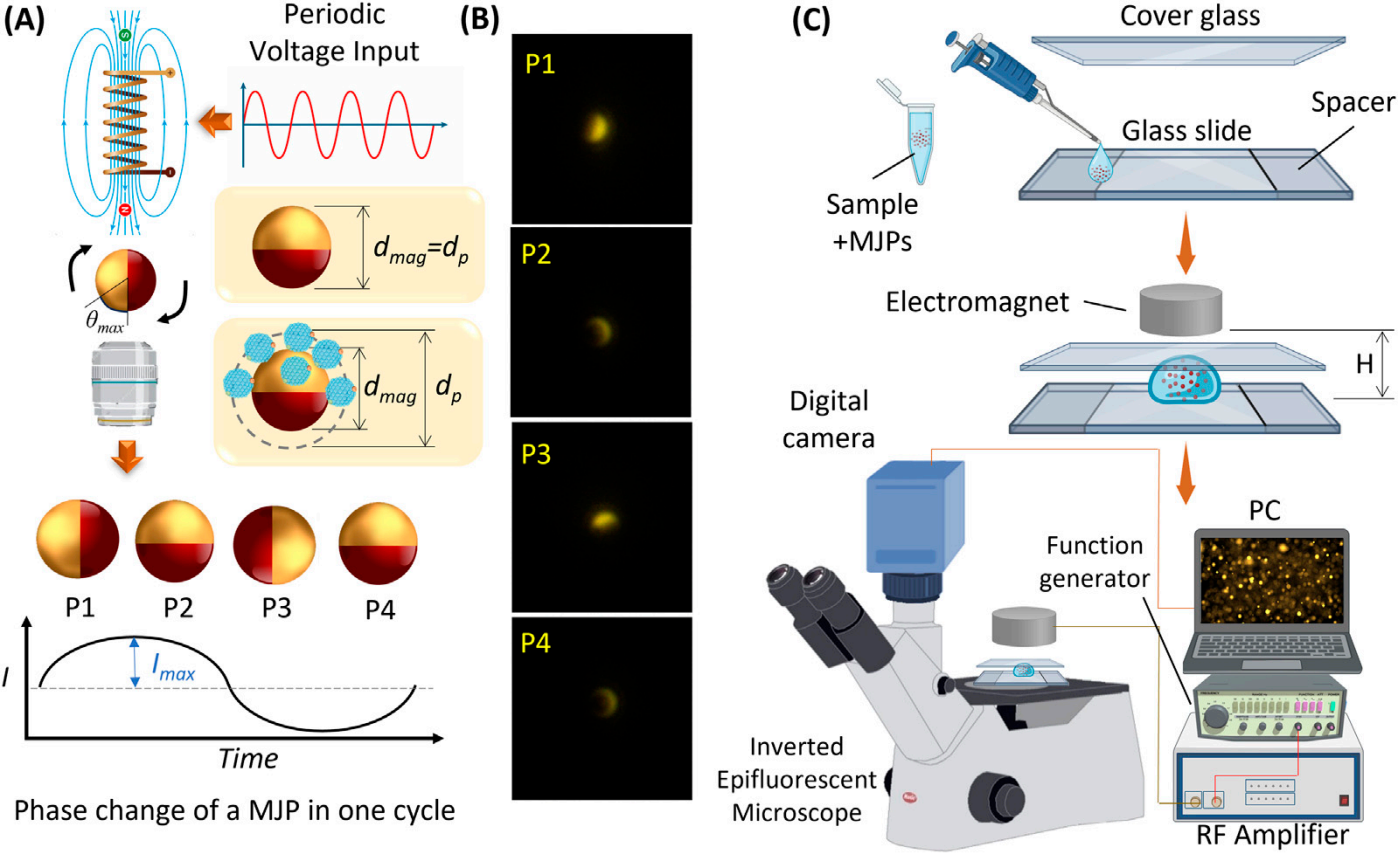
**(A)** The mechanism of MJP manipulation with an electromagnet. The rotation of MJP is modulated by an electromagnet with its periodically switching polarity. Periodic blinking signal is then measured from the rotating MJP. **(B)** Serial images exhibit four major phases of an MJP under one cycle of a periodic signal. The four phases correspond to the sinusoidal signal shown in **(A)**. **(C)** Schematic of an MJP platform's experimental setup. The top exploded diagrams exhibit the sample loading and deployment of sandwiched glass slides and electromagnet.

The mechanism of MJP manipulation is illustrated in Figure 1A. The magnetic polarity switching of the electromagnet was implemented using an electrical current with a square waveform and 50% duty cycle. The major advantages of a nonmoving magnetic field over their prior counterparts (Sinn et al., 2011; Li et al., 2014) are reduced mechanical vibration and capability to manipulate MJPs at high driving frequencies (Figure 1B). The maximum rotational angle of MJPs in a cycle can be expressed as follows (Sinn et al., 2011):

$${∆\theta }_{\max}=\frac{\chi^{\prime \prime }V_{m}B^{2}}{\kappa \eta fV_{H}\mu_{0}},\left({∆\theta }_{\max}\leq \pi \right),$$ (1)

Where *χ*″ is the imaginary part of magnetic susceptibility (which is magnetic content dependent), *V*_*m*_ is the volume of the bead’s magnetic content (which is proportional to the surface area of the bead), *B* is the magnetic field strength, *μ*_0_ is the permeability of free space, *η* is the fluid viscosity, *f* is the driving frequency, *V*_*H*_ is the hydrodynamic volume of the MJP, and *κ* is the shape factor of the bead (*κ* = 6 for a sphere). The maximum rotational angle Δ*θ*_*max*_ with respect to the driving frequency decreases as the fluid viscosity or the effective volume of the MJP escalates. Increase in drag compromises MJPs’ capability to follow the external magnetic field. Notably, the MJPs’ effective volume increased by captured sEVs slows down the MJPs. However, the MJPs’ effective volume increased by the growth of core PS particles intensifies the magnetic effect, enhancing tracking capability. Thus, the cutoff frequency, that is, the critical driving frequency that MJPs stop to follow the external magnetic field, can be a good indicator of angular change in beads. The blinking signal measured from each rotating MJP is expressed in terms of fluorescent intensity. The time-dependent intensity is formulated as follows:

$$I=A\frac{d_{mag}^{2}}{4}{∆\theta }_{\max}\left[\sin\left(2\pi ft+\emptyset \right)+1\right],$$ (2)

Where *d*_*mag*_ is the core particle diameter, ∅ is the initial phase, and *A* is a constant of the amplitude. The intensity progressively decreased with the value of Δ*θ*_*max*_, which is a function of the particle’s magnetic moment, magnetic field strength, fluid viscosity, driving frequency, and effective volume of MJPs. For instance, *I* decreases rapidly at a driving frequency *f* scan as fluid viscosity increases. Notably, the magnetic content is subject to *d*_*mag*_. *d*_*p*_, which is here defined as the overall particle diameter, is equivalent to *d*_*mag*_ when no external objects attach to the MJPs (Figure 1A). To quantify the blinking signal, the wavelet algorithm was employed. The wavelet algorithm transformed a time-dependent signal waveform into a frequency spectrum plot. Subsequently, a relationship between spectrum intensity and driving frequency was obtained from the frequency spectrum. Signal intensity (SI) is defined as a ratio of the maximum intensity at the driving frequency to the lowest background noise level in a frequency spectrum plot. The final cutoff frequency is determined when the signal becomes undetectable. The ideal SI threshold is theoretically set to 1, but in practice, background noise and resolution limits typically elevate the actual SI threshold. According to Equation 2, the cutoff frequency is strongly associated with the particle’s magnetic moment, magnetic field strength, fluid viscosity, driving frequency, and effective volume of the MJP. The subtle particle or fluid conditions can be quantitatively obtained by judging the cutoff frequency of MJPs with the proposed electromagnet system.

For measurement (Figure 1C), functionalized MJPs were first mixed with a sample solution to be detected. Next, 2 μL of MJP suspension was pipetted to a glass slide, and then a cover glass was placed on top of the glass slide. The cover glass and glass slide were separated by a spacer composed of three layers of 3 M tapes stacked up to 165 μm. An electromagnet (ZYE1-P20/15, Keyes, China) was positioned above the suspension with a fixed distance to impose a decent magnetic field over the MJPs. The electromagnet was driven by a periodic function generator (GFG-3015, GW INSTEK, Taiwan) amplified by a power amplifier (Model 234, TEGAM, OH, United States), and the driving frequency was sequentially modulated from 3 Hz to 22 Hz. The glass substrate was placed on an inverted epifluorescent microscope (IX73, Olympus, Japan), and the MJPs were visualized with a 20× objective (0.45 NA, Olympus, Japan) and a fluorescent filter cube (Ex:530-550/DM:570/EM:575; U-FF, Olympus, Japan). Subsequently, particle images were recorded for 10 s with a fast digital camera (BFS-U3-23S3C-C, FLIR, Canada) at a frame rate of 80 fps.

## 3 Results and discussion

### 3.1 Fluid viscosity, magnetic field strength, and particle diameter changes predicted with simulated images

**FIGURE 2:**
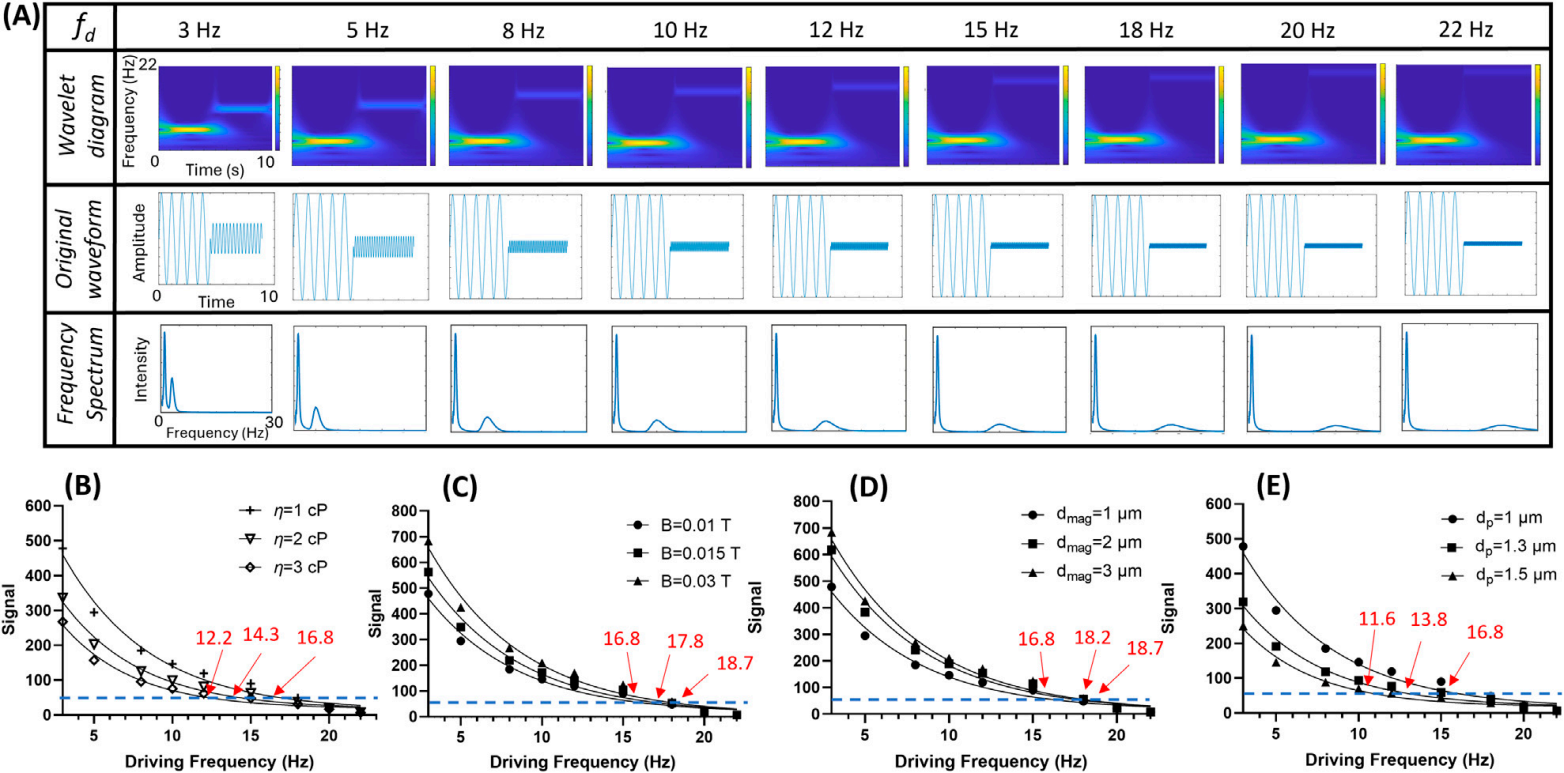
Theoretical predictions with simulated images. **(A)** An example of wavelet transform scalogram (top row) and frequency spectrum (bottom row) derived from the simulated signal waveforms (middle row) from 3 Hz to 22 Hz with 80 fps. **(B)** Scanned signal intensities (SIs) at three different fluid viscosity (*η* = 1, 2, and 3 cP) and their cutoff frequencies (threshold = 50). **(C)** Scanned SIs of three magnetic field strength (B = 0.01, 0.015, and 0.03 T) and their cutoff frequencies (threshold = 50). **(D)** Scanned SIs of three different core particle diameters (*d*_*mag*_ = 1, 2, and 3 μm) and their cutoff frequencies (threshold = 50). Noted that the overall particle diameter *d_p_* is identical to *d_mag_* in this case. **(E)** Scanned SIs of three different overall particle diameters (*d*_*p*_ = 1, 1.3, and 1.5 μm) with their PS core fixed at *d*_*mag*_ = 1 μm and their cutoff frequencies (threshold = 50).

The performance of the theoretical behavior of the MJPs was investigated. Changes in magnetic field strength, fluid viscosity, and effective volume of the MJPs after the capturing of sEVs or growth of core PS particles were individually investigated using the algorithms expressed in Equations 1, 2. Notably, some MJPs may not respond to an external magnetic field because of microfabrication defects. Therefore, a driving frequency of 1 Hz was used before each measurement for the screening of responsive MJPs. The MJPs without a 1 Hz frequency signature were discarded. This screening step can save processing time and increase accuracy. Moreover, a synthetic waveform composed of a low frequency fixed at 1 Hz and a high frequency varying from 3 Hz to 22 Hz was employed under simulation and experimental conditions. In the theoretical evaluation, a series of synthetic waveforms (middle row, Figure 2A) were generated according to Equation 2 using MATLAB for simulating an anticipated condition (*V*_*m*_, *χ*″, *B*, *η*, *V*_*H*_). For each driving frequency, the waveform consists of a 1-Hz sinusoidal wave and a high-frequency sinusoidal wave identical to the driving frequency. Each sinusoidal wave lasts for 5 s, resulting in a total duration of 10 s for the synthetic waveform. Two-dimensional (2D) wavelet transform scalograms were therefore derived from the abovementioned synthetic waveforms after being analyzed with the wavelet algorithm (top row, Figure 2A). Two bars corresponding to the two driving frequencies can be clearly observed in all the wavelet diagrams. When the low-frequency bar was fixed at 1 Hz, the high-frequency bar increased as the driving frequency increased from 3 Hz to 22 Hz. The total time required for the operation was approximately 90 s. By summing up the wavelet transform scalograms over time, a 2D plot showing the frequency spectrum with respect to the driving frequency was obtained (bottom row, Figure 2A). The SI decreased with driving frequency, as predicted by Equation 1. In addition, the SI declination rate tended to vary with environmental conditions, such as the magnetic moment of the MJPs, magnetic field strength, fluid viscosity, and effective volume of the MJPs, as stated in Equation 2. For validation, 800 simulated images with a frame rate of 80 fps according to Equation 1 were generated for each condition. The cutoff SI was empirically set at 50 (i.e., the bar at the higher driving frequency became unrecognizable in the wavelet diagram).

Evaluation was performed at viscosity (*η*) of 3, 2, and 1 cP, which correspond to cutoff frequencies of 12.2, 14.3, and 16.8 Hz, respectively (Figure 2B). The increased viscosity appeared to impede the MJPs from following the magnetic field with high drag. A high magnetic field strength or a high particle magnetic moment enabled the MJPs to follow the magnetic field, increasing the cutoff frequency. As a result, at tested magnetic field strength (*B*) of 0.01, 0.02, and 0.03 T, the cutoff frequencies were 16.8, 17.8, and 18.7 Hz, respectively (Figure 2C). On particle diameter factor, two circumstances were investigated. The first case was MJP diameter change due to the growth core PS particles, and the second case was MJP diameter change due to the attachment of targets to the beads’ surfaces. In the first case, the magnetic content (Ni) increased with the growth of the core PS particles. As a result, *V*_*m*_ and *χ*″ increased with nickel coating area ( $\propto d_{mag}^{2}$ ). Therefore, the cutoff frequency increased because the SI increased with MJP size (Figure 2D). By contrast, in the second case, the magnetic content remained the same even though the overall volume of the MJP increased (Figure 2E). The increased MJP diameter mainly originated from the attached targets. As a result, the cutoff frequency decreased considerably because the mobility of the MJPs was impeded by drag. The results indicated that the MJPs can be used as biosensors for the dose-dependent monitoring of different environmental conditions.

### 3.2 Effects of screened and unscreened MJP blinking signals

**FIGURE 3:**
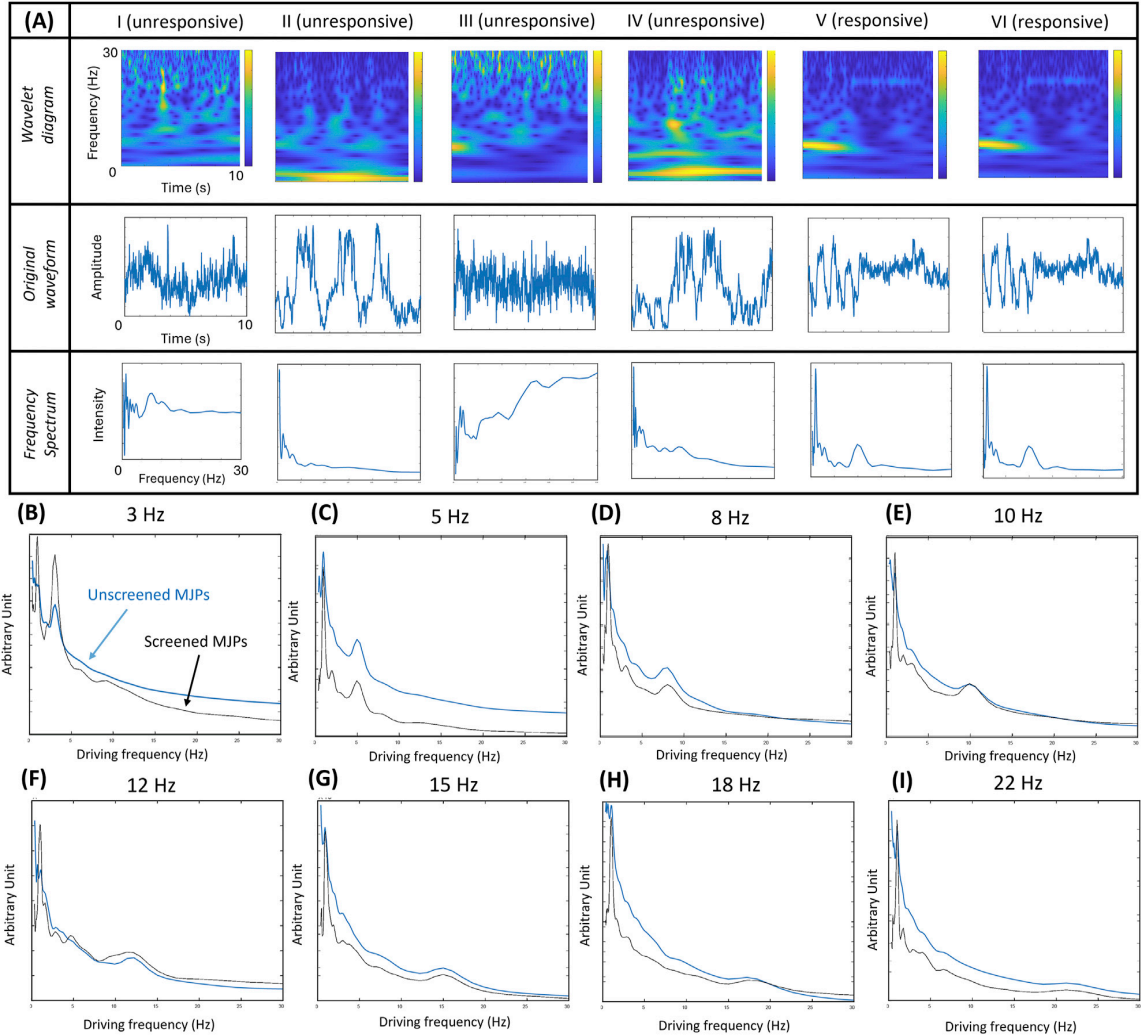
**(A)** Wavelet transform scalograms, signal waveforms, and frequency spectra of representative MJPs. Particles I–IV refer to unresponsive MJPs and particles V–VI refer to responsive MJPs. **(B-I)** Driving frequency scans of screened MJPs (V–VI) in blue color and unscreened MJPs (I–VI) in black color from 3 Hz to 22 Hz. With increased random background noise, the unscreened MJPs show higher background levels and less significant frequency peaks.

The MJPs were carefully sorted by size before they were used for biosensing applications. The procedure is detailed in the *Methods and Materials* section. However, some defective MJPs, such as those unresponsive to the external magnetic field, may have remained in the suspension. The likely causes were coating failure, insufficient or excessive metal coverage, and presence of trimers or dimers. To prevent interferences from defective MJPs, a signature of 1 Hz driving frequency was incorporated during manipulation for the sorting of responsive MJPs out from the total particle population. The performance of the screened and unscreened MJP blinking signals was evaluated using the images of 1-μm MJPs obtained at an input voltage of 10 V_pp_ and an electromagnet, which was 3.6 cm away from the glass slide, at 80 Hz for 10 s under a 20× objective. A total of 127 particles were tracked and analyzed. Some representative images at a driving frequency of 10 Hz, including their wavelet transform scalograms, signal waveforms, and frequency spectra, are depicted in Figure 3A. Images I–IV represent unresponsive MJPs, whereas images V–VI stand for responsive MJPs. The signal may be unrecognizable without screening (i.e., images I–VI) because it is often mixed with noise. Conversely, a signal peak corresponding to the driving frequency can be easily identified in screened cases (i.e., images V–VI). Based on the principle, driving frequency scanning was conducted from 3Hz to 22 Hz. The frequency spectra of the screened and unscreened signals indicated that screened MJPs can show more consistent and stable spectra than the unscreened MJPs (Figures 3B–I). For example, the peak at 10 Hz from the unscreened MJPs nearly disappeared (Figure 3E). However, a peak obtained from the screened MJPs was easily observed. Hence, the screening procedure can effectively mitigate uncertainty in the results.

**FIGURE 4:**
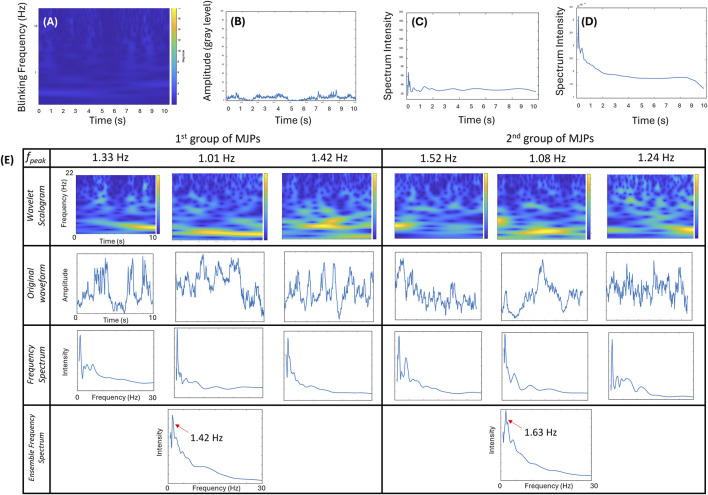
Effect of MJPs controlled under a driving frequency of 0 Hz. **(A–C)** Wavelet transform scalogram, signal waveform, and frequency spectrum of a representative MJP are exhibited. **(D)** Ensemble frequency spectra (*n* = 42 particles). No signal peak except the low-frequency pedestal is observed in the plot. **(E)** Effects of free-suspending MJPs measured from two independent groups. Wavelet transform scalograms (1st row), signal waveforms (2nd row), and frequency spectra (3rd row) of two groups of representative MJPs are exhibited. Ensemble frequency spectra over 65 MJPs per group are depicted. Significant peaks (left: 1.42 Hz; right: 1.63 Hz in the 4th row) are explicitly observed.

Apart from MJP screening, responsive MJPs were evaluated for their susceptibility to diffusion given that Brownian motion and active magnetic manipulation likely have overlapping signals at low frequencies (∼1–2 Hz). For clarification, 1-μm MJPs were trapped in a medium by an electromagnet at 0 Hz, and their blinking signals were recorded for 15 s. No discernible frequency bands were observed in the wavelet transform scalogram (Figure 4A), implying the static state of the MJPs. Similarly, the corresponding waveform of the blinking signals (Figure 4B) and frequency spectrum (Figure 4C) showed insignificant and random variations, compared with their counterparts at other driving frequencies (Figure 3). The ensemble frequency spectrum of the overall particle images showed no major peaks between 1 and 25 Hz (Figure 4D). Conversely, the same MJPs appeared to have restored Brownian motion right after the electromagnet was switched off. Some representative wavelet transform scalograms, waveforms, and frequency spectra are depicted in Figure 4E. The peak frequencies measured from different MJPs ranged roughly between 1 and 2 Hz. Ensemble frequency spectra from two separate groups were estimated. The results showed that their individual peak frequencies fell at 1.42 and 1.63 Hz, implying that the electromagnet was necessary to the manipulation of MJPs and prevented blinking signals from undergoing serious cross talks with background Brownian motion.

### 3.3 Experimental evaluations of the cutoff frequencies

**FIGURE 5:**
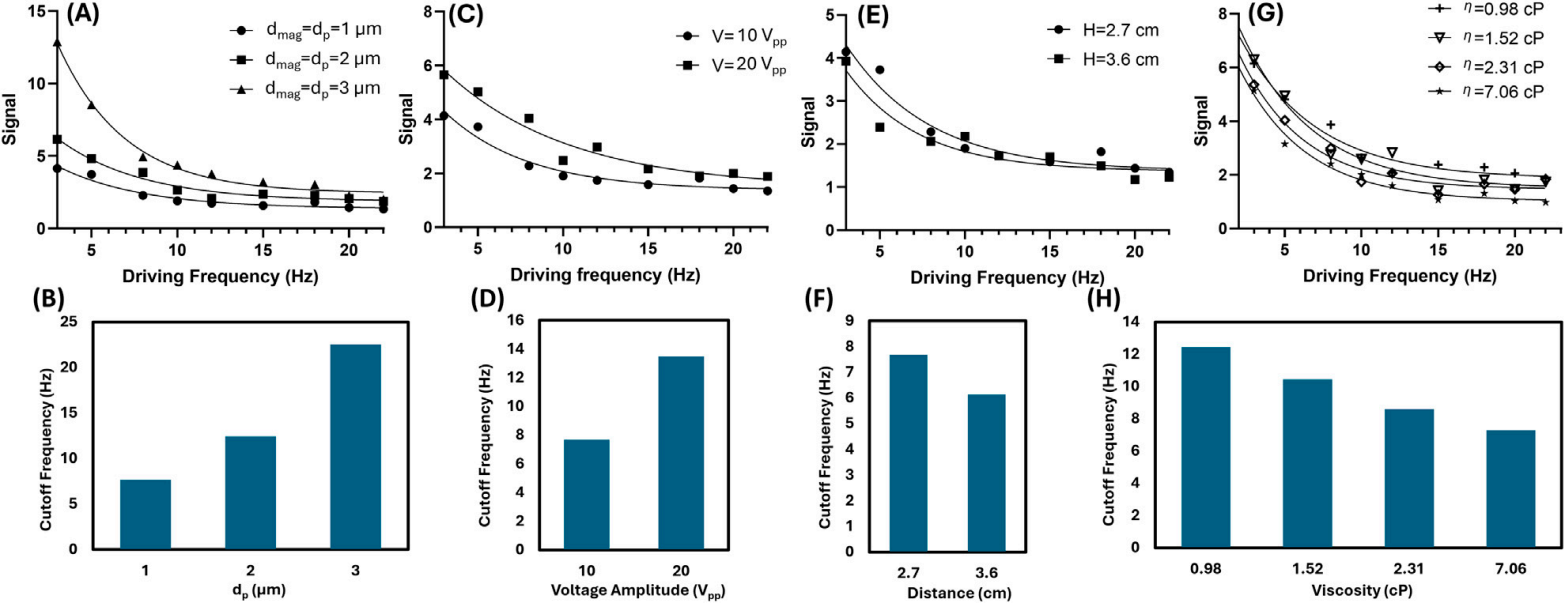
Cutoff frequencies in response to particle diameter, input voltage, distance, and fluid viscosity. **(A,B)** *f*_*cutoff*_ = 7.7, 12.4, and 22.6 Hz corresponding to the core particle diameters *d*_*p*_ = *d*_*mag*_ = 1, 2, and 3 μm, respectively. **(C,D)** *f*_*cutoff*_ = 7.7 and 13.5 Hz corresponding to the input voltages *V*_*input*_ = 10 and 20 V_pp_, respectively with all their particle diameters fixed at *d_p_* =*d_mag_* = 1 μm. **(E,F)** *f*_*cutoff*_ = 7.7 and 6.1 Hz corresponding to the distances *H* = 2.7, 3.6 cm, respectively with all their particle diameters fixed at *d_p_* =*d_mag_* = 1 μm. **(G,H)** *f*_*cutoff*_ = 12.4, 10.4, 8.6, and 7.3 Hz corresponding to the fluid viscosities *η* = 0.98, 1.52, 2.31, and 7.06 cP, respectively with all their particle diameters fixed at *d_p_* =*d_mag_* = 2 μm.

The MJP-enabled biosensing capabilities predicted by the abovementioned theory expressed in Equations 1, 2 were investigated through proof-of-concept experiments on particle diameter, input voltage, distance, and fluid viscosity. Regarding particle diameter, the cutoff frequency increased with core PS particle diameter, consistent with the simulation results (Figures 5A,B; detailed data shown in Supplementary Figures S4, S5). Notably, a large MJP contributed to high m and *χ*″ with increased magnetic content. Therefore, the change in intensity was proportional to particle diameter. Based on the relationship, large MJPs led to a higher cutoff frequency. While the ideal SI threshold is theoretically defined as 1, herein the SI threshold was empirically determined to be 2.5 for all experimental measurements due to background noise and resolution limits. Regarding voltage and distance, both cases altered the magnetic field strength. Thus, a high voltage (Figures 5C,D; detailed data shown in Supplementary Figures S4, S6) or a short distance (Figures 5E,F; detailed data shown in Supplementary Figures S4, S7) promoted the SI, resulting in a high cutoff frequency and *vice versa*. According to Equation 1, the SI decreases with the fluid viscosity. The fluid viscosity was adjusted by mixing deionized (DI) water and glycerol. Four different ratios of glycerol solutions from low to high were prepared to yield viscosity of 0.98, 1.52, 2.31, and 7.06 cP, and the cutoff frequencies from low to high reached 12.4, 10.4, 8.6, and 7.3 Hz, respectively. The experimental data agreed well with the predicted trend (Figure 2B), showing that a high viscosity can slow down cutoff frequency because of high drag (Figures 5G,H; detailed data shown in Supplementary Figures S4, S8). The cutoff frequency represents a statistical result derived from the ensemble average of multiple MJPs analyzed in the recorded particle image. The numbers of responsive MJPs counted are detailed in Supplementary Figures S4–S8. Repeatability was evaluated alongside other tests. Six independent measurements of 2-µm plain MJPs suspended in DI water (*η* = 0.98 cP) yielded a cutoff frequency of 12.9 ± 0.5 Hz Supplementary Figure S9, corresponding to a 3.87% variation.

### 3.4 Validation of the MJP system with the sEV detection

**FIGURE 6:**
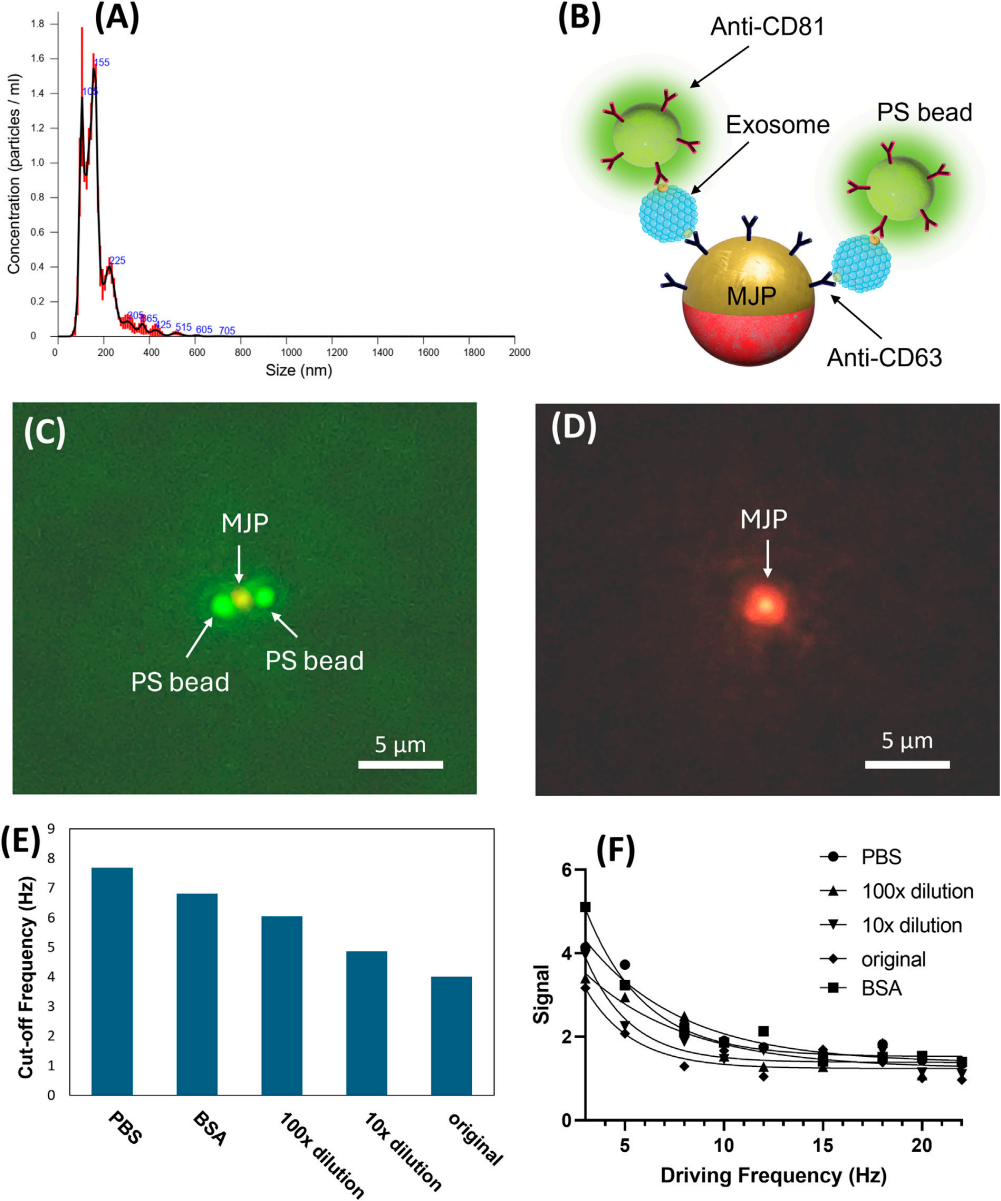
**(A)** NTA analysis for the size distribution of the isolated sEVs. **(B)** Schematic of the exosome detection. The 0.2 µm green fluorescent PS beads were used to confirm the colocalization configuration. **(C)** Microscopic image of the successful colocalized MJP and PS beads. **(D)** The middle MJP was reconfirmed by switching to a green filter cube (Ex:530-550/DM:570/EM:575). **(E)** Signal intensities scanned over a range of frequencies from 3Hz to 22 Hz for different medium conditions. **(F)** *f*_*cutoff*_ = 7.7, 6.8, 6.0, 4.9, and 4.0 Hz corresponded to the medium conditions PBS, BSA, 100× dilution, 10× dilution, and original isolated exosomes, respectively. The cutoff frequency decreases linearly for the last three exosome cases as their concentration logarithmically increases.

After the establishment of the proposed MJP system, a proof-of-concept validation was conducted to demonstrate practicability. Given that the sEV size was similar to our MJP and effective in promoting considerable volumetric change, the sEV isolates obtained from the cell culture medium of the OECM-1 cell line were employed. The NTA report showed that the major peak of the sEV size distribution was 169.5 ± 3.4 nm and the concentration was 2.9 × 10^11^ particles mL^−1^ (Figure 6A). The MJP-sEV immunocomplex was verified using a mock immunocomplex configured with a 1-μm MJP, sEVs, and 200-nm green fluorescent PS beads (Figure 6B). The successful formation of the anticipated immunocomplex (Figure 6C) was visualized using the green fluorescent PS particles. Under a green filter, the middle MJP of the same immunocomplex in Figure 6C was reconfirmed (Figure 6D). Notably, the fluorescent images did not reflect the physical size of the particles because of optical diffraction. Dose-dependent effects of OECM-1-secreted sEVs on the MJP system were investigated. Three concentrations were prepared for the test after tenfold serial dilution with the 1× PBS: undiluted (original concentration), tenfold dilution, and 100-fold dilution. PBS served as a control, whereas 0.1% BSA was used in determining non-specificity. The result showed that the cutoff frequency decreased with the sEV concentration (Figures 6E, F). When the sEV solutions were diluted one-, ten-, and 100-fold, the cutoff frequencies were 4.0, 4.9, and 6.0 Hz, respectively, which remained lower than the cutoff frequency in the control (PBS buffer, *f*_*cutoff*_ = 7.7 Hz). The BSA group showed a lower cutoff frequency (*f*_*cutoff*_ = 6.8 Hz) than the control (*f*_*cutoff*_ = 7.7 Hz) and thus likely had higher viscosity than PBS buffer. Notably, the specificity between the BSA and all other sEV groups was distinguishable. Considering the best detectable concentration was observed in the 100-fold dilution group, the minimal detected concentration for sEVs in the current study was eventually determined to be 2.9 × 10^9^ particles mL^−1^.

## 4 Conclusion

sEVs have emerged as potential cancer biomarkers in recent years. However, their small physical size, vulnerability, and trace amounts in small samples pose considerable barriers to POCT diagnosis. To address the challenges, we developed an active MJP system to detect the presence of cancer cell OECM-1-secreted sEVs, which carry specific surface biomarkers. For characterization, the newly developed MJP system was evaluated theoretically and experimentally. To improve the signal-to-noise ratio, a 1 Hz driving frequency was employed in the measurement, and nonresponsive MJPs were excluded. The result showed that the images acquired from sorted particles tended to provide consistent data for analysis. Theoretically, the predicted cutoff frequency increased with increasing magnetic moment, magnetic field strength, and core particle size, and decreasing fluid viscosity. Experimentally, the measured cutoff frequencies showed good agreement with the theoretical predictions. Particle size may vary in two ways. When the core PS particle grows, the cutoff frequency increases owing to increase in magnetic content. However, when the MJP diameter increases because of captured sEVs, the cutoff frequency decreases even when the core PS particle remains. The anticipated immunocomplex was successfully visualized with fluorescent particles and colocalized sEVs. In addition, dose-dependent detection and specificity were investigated. The minimum detected concentration of sEVs reached 2.9 × 10^9^ particles mL^−1^, and the specificity of the sEV markers, including CD63, was acceptable. Based on this proof-of-concept evidence, follow-up studies expanding on various sEVs surface biomarkers, cancer cell lines, and human clinical samples will be conducted in replicates to confirm the practical feasibility and identify the LoD of the MJP system. The primary limitation of the current MJP technique stems from its microfabrication challenges. While smaller MJPs could enhance sensitivity, the current fabrication process restricts MJP sizes to larger than 1 μm due to low yields for sub-micron MJPs. Additionally, the hemispherical coating of Au/Ni/Ag varies between MJPs, potentially causing non-uniformity in the blinking signal. Addressing these two concerns could further improve the reported minimum detected concentration in our study, enhancing the method’s overall performance, efficiency, and sensitivity. Overall, this study presents a promising diagnostic tool that may be applied to future POCT applications.

## Data Availability

The raw data supporting the conclusions of this article will be made available by the authors, without undue reservation.
