# Supplementary figures and images for: Periodic blinking manipulation of magnetic Janus particles with a tunable electromagnetic field for rapid sensing of extracellular vesicles
Source: Front Bioeng Biotechnol. 2025 Apr 25;13:1565479. doi: 10.3389/fbioe.2025.1565479 (PMC12062064; doi:10.3389/fbioe.2025.1565479)

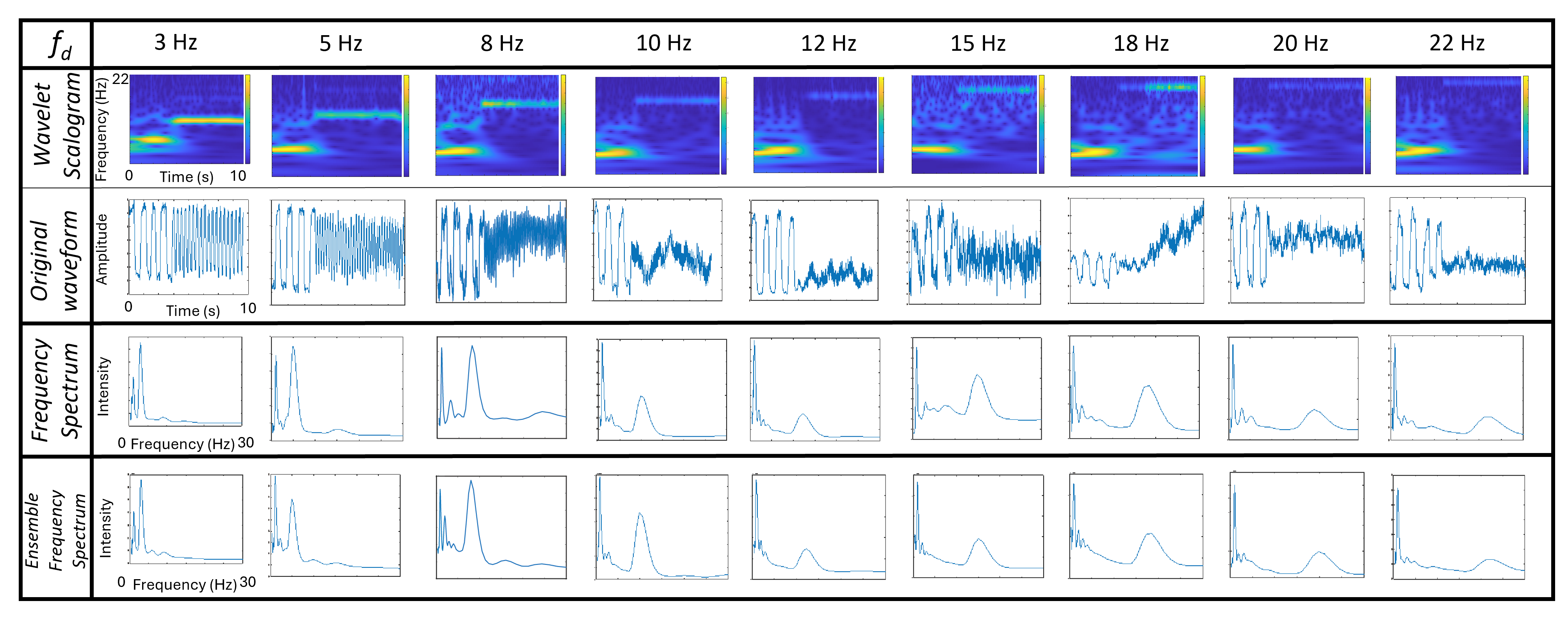

Supplement: Supplementary file 1 [file Image6.tif]

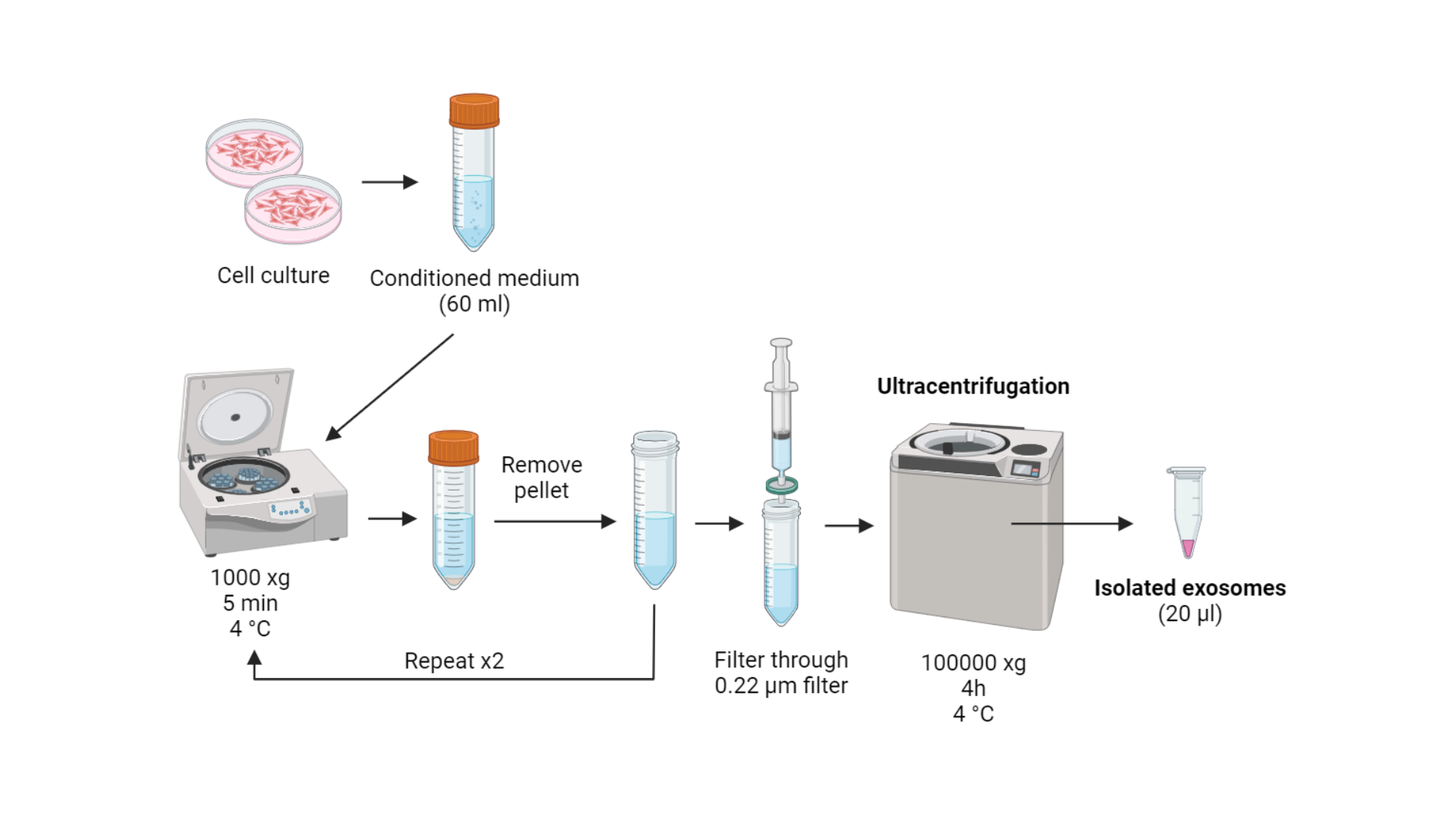

Supplement: Supplementary file 2 [file Image3.tif]

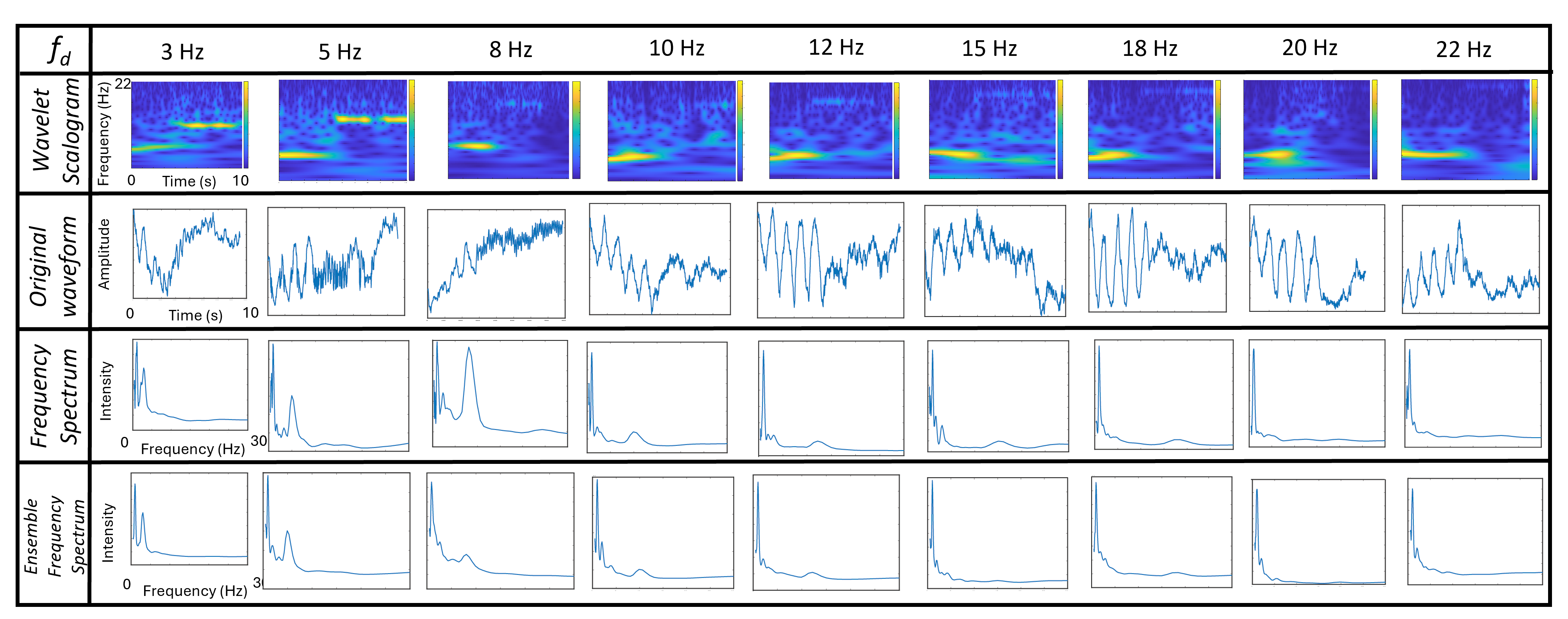

Supplement: Supplementary file 3 [file Image4.tif]

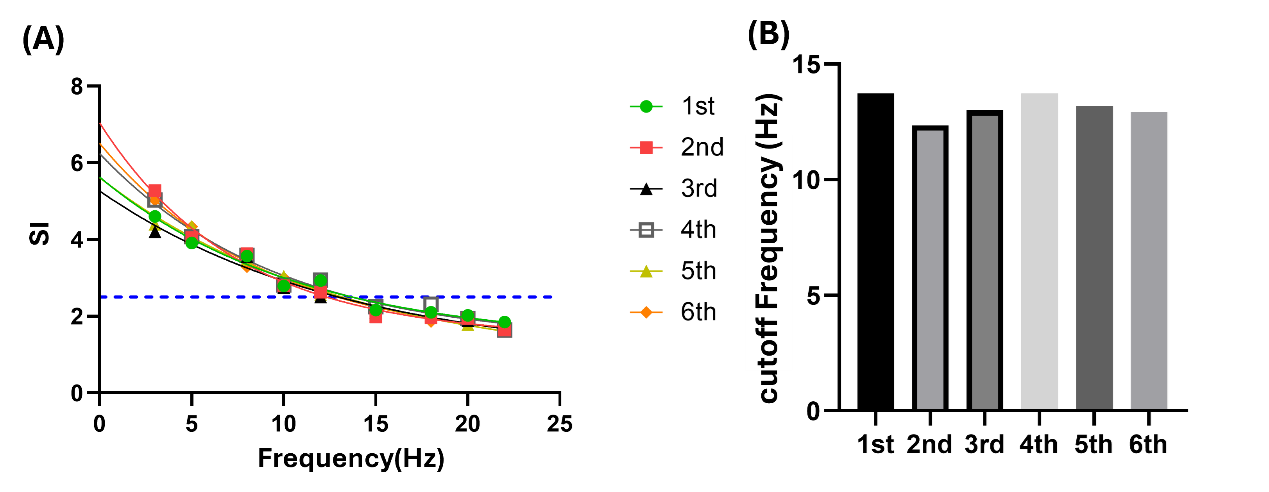

Supplement: Supplementary file 4 [file Image9.tif]

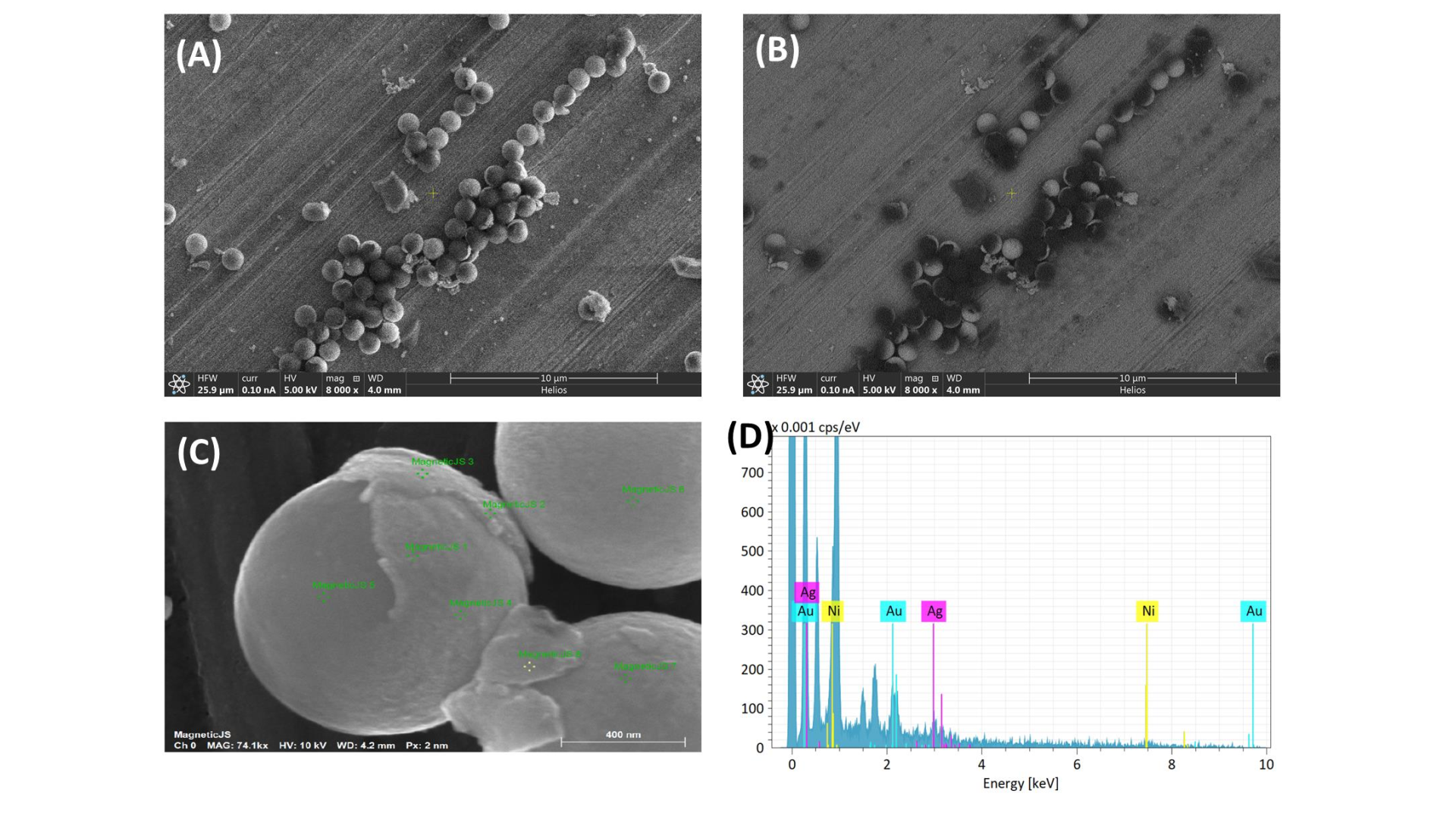

Supplement: Supplementary file 5 [file Image2.tif]

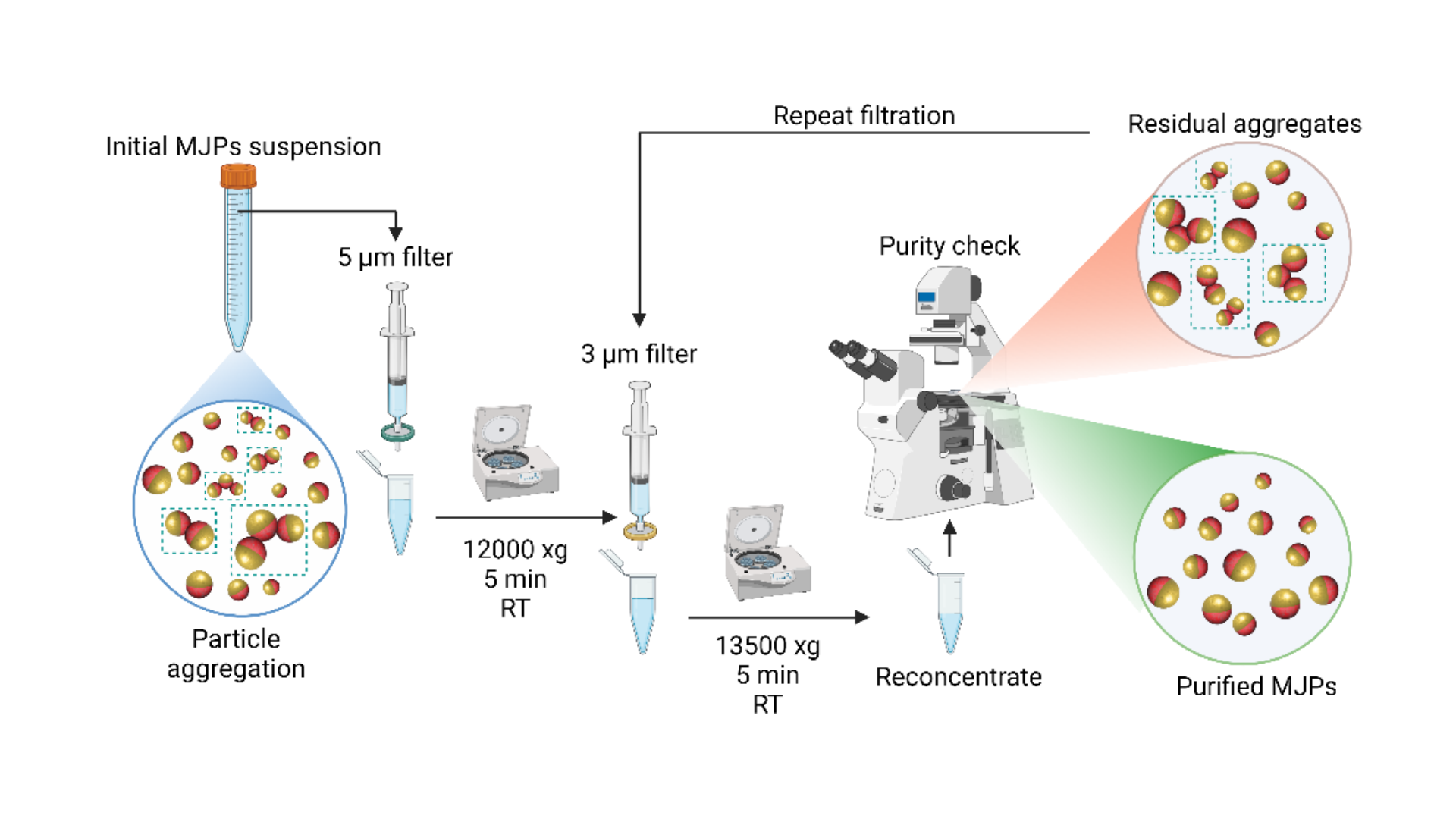

Supplement: Supplementary file 6 [file Image1.tif]

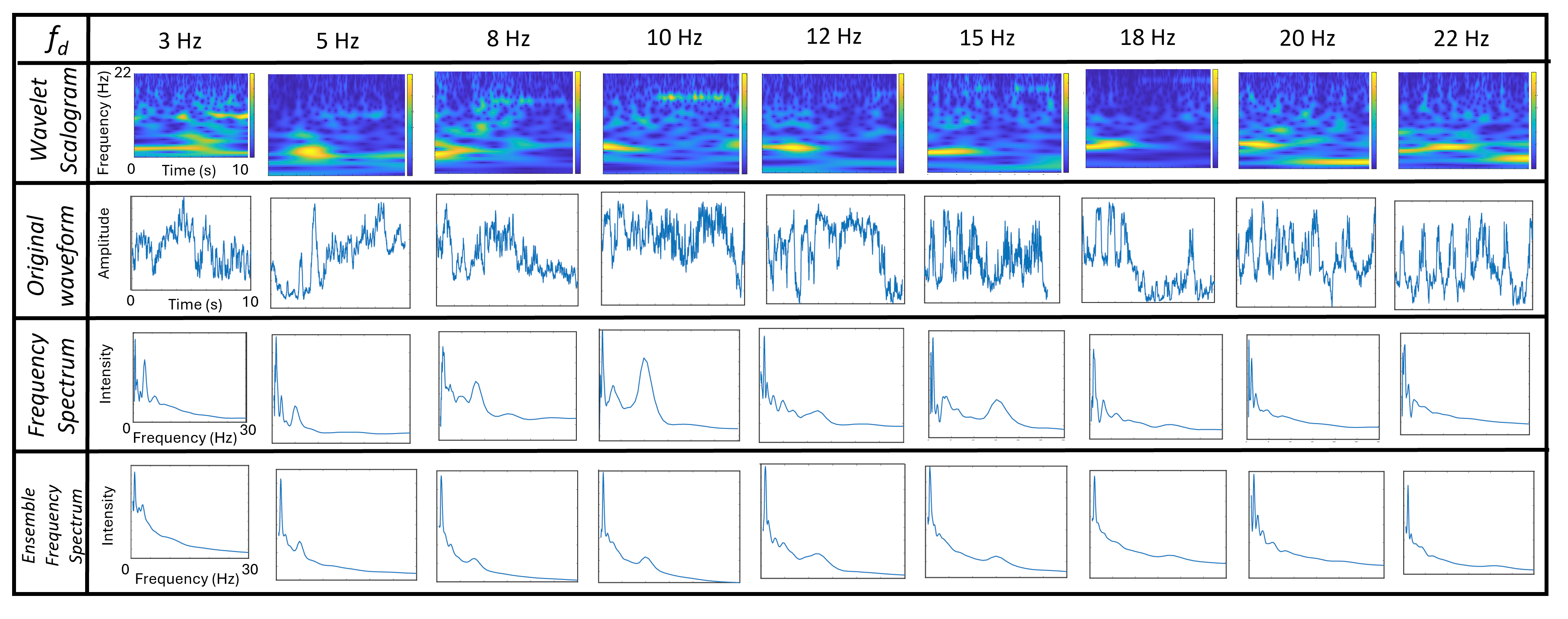

Supplement: Supplementary file 7 [file Image7.tif]

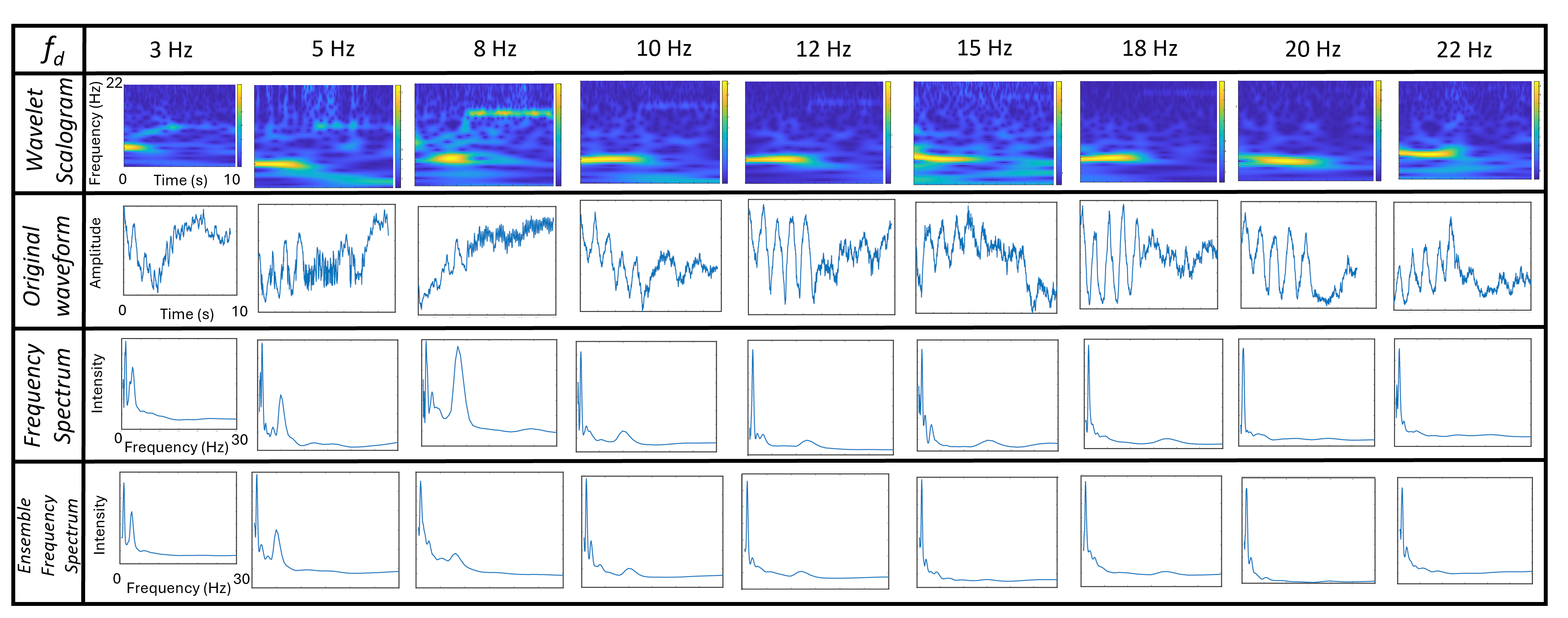

Supplement: Supplementary file 8 [file Image8.tif]

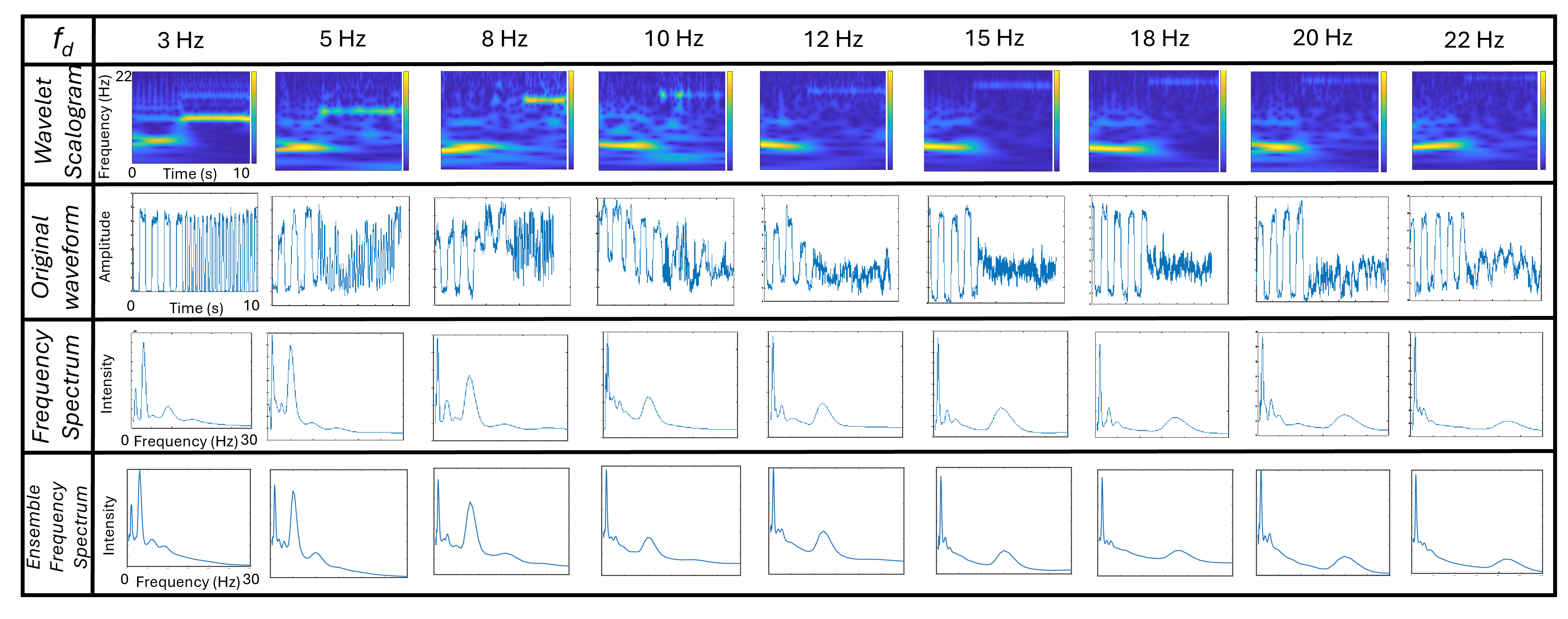

Supplement: Supplementary file 9 [file Image5.tif]
