# Supplementary material for: Periodic blinking manipulation of magnetic Janus particles with a tunable electromagnetic field for rapid sensing of extracellular vesicles
Source: Front Bioeng Biotechnol. 2025 Apr 25;13:1565479. doi: 10.3389/fbioe.2025.1565479 (PMC12062064; doi:10.3389/fbioe.2025.1565479)
Supplement: Supplementary file 11 [file DataSheet1.docx]

**Periodic Blinking Manipulation of Magnetic Janus Particles with a Tunable Electromagnetic Field for Rapid Sensing of Extracellular Vesicles**

**Han-Sheng Chuang^1, 2, *^,** **Thi Thanh Huong Pham^1^, Yu-Hsuan Chou^1^, Chi-Ying F. Huang^3^, Ting-Yuan Tu^1^, Tai-Hua Yang^1, 4^, Jhih-Cheng Wang^5, 6, 7 *^**

^1^Department of Biomedical Engineering, National Cheng Kung University, Tainan, Taiwan

^2^Medical Device Innovation Center, National Cheng Kung University, Tainan, Taiwan

^3^Institute of Biopharmaceutical Sciences, National Yang Ming Chiao Tung University, Taipei, Taiwan

^4^Department of Orthopedic Surgery, National Cheng Kung University Hospital, Tainan, Taiwan

^5^Department of Urology, Chimei Medical Center, Tainan, Taiwan

^6^Department of Electrical Engineering, Southern Taiwan University of Science and Technology, Tainan, Taiwan

^7^School of Medicine, College of Medicine, National Sun Yat-sen University, Kaohsiung, Taiwan

*** **Correspondence**

[*oswaldchuang@mail.ncku.edu.tw*](mailto:oswaldchuang@mail.ncku.edu.tw)

[*tratadowang@gmail.com*](mailto:tratadowang@gmail.com)

***Supplementary Material***

**
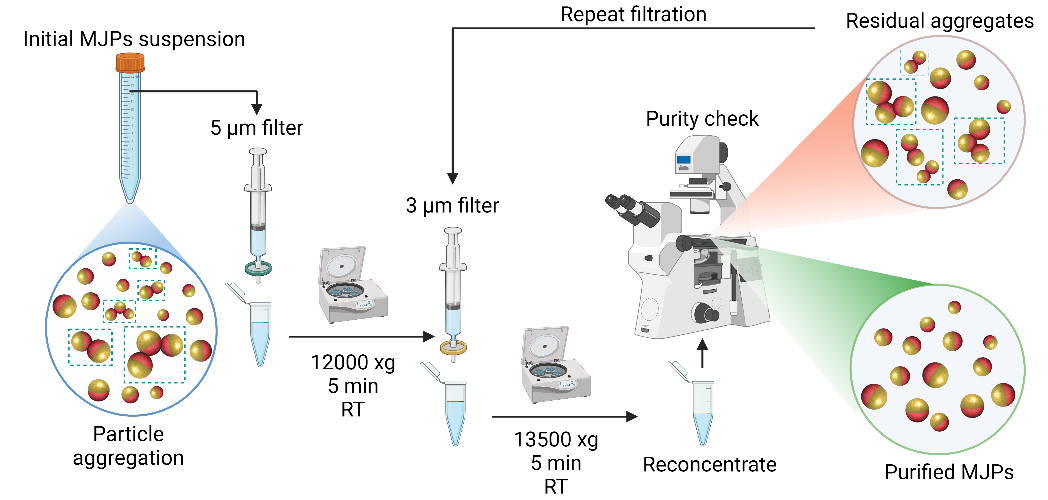
**

**Supplementary Figure 1.** Flow chart of two-step MJP purification. First step: the collected MJPs from the glass slide were transferred to 5 μm filter disk and subjected to 5 min of centrifugation. Second step: the filtrate from the first step was injected into another 3 μm filter disk and subjected to 5 min of centrifugation.


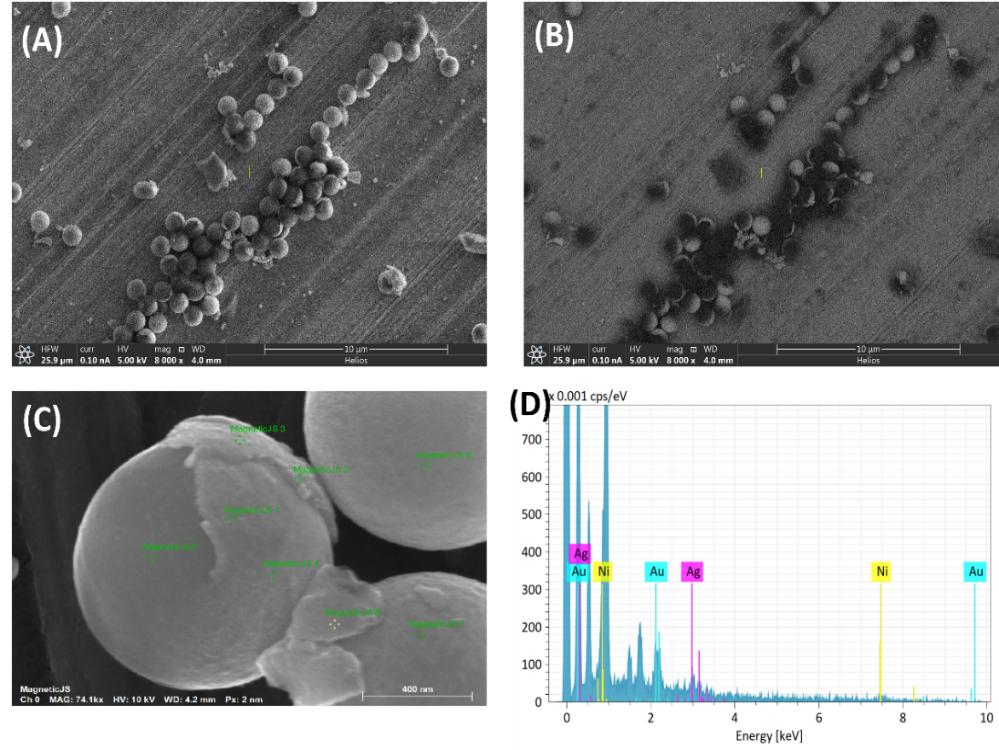


**Supplementary Figure 2.** (A) Secondary electrons SEM image of MJPs and (B) backscattered electrons SEM image of the same MJPs. The bright shells indicate where the metallic coatings are. (C) Scanning of the surfaces of MJPs with energy dispersive X-ray spectrometer (EDS). The green marks indicate where the elements are analyzed. (D) EDS analysis for those elements covering the MJPs. Gold, silver, and nickel are the three major elements of the coating.


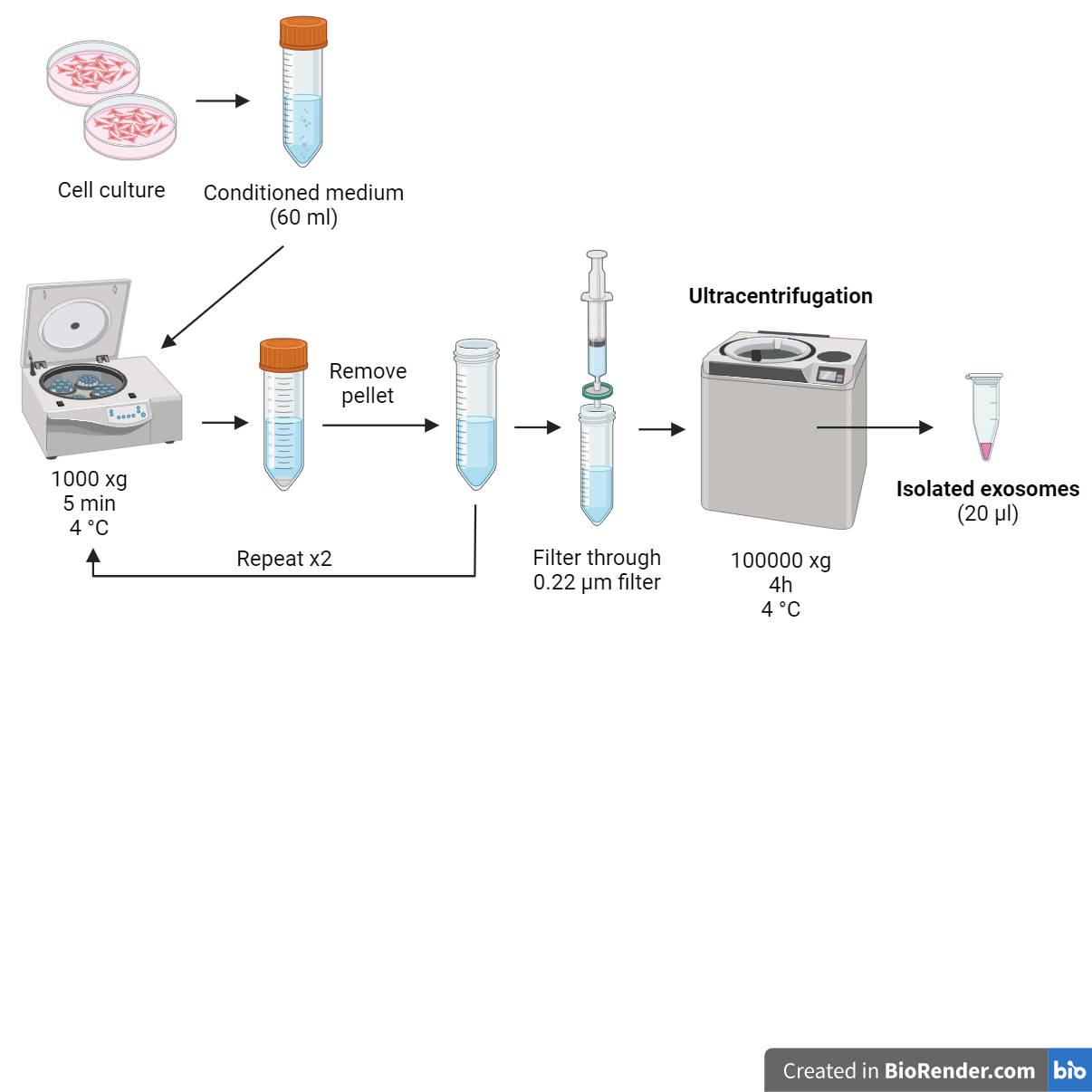


**Supplementary Figure 3.** Flow chart of sEV isolation.


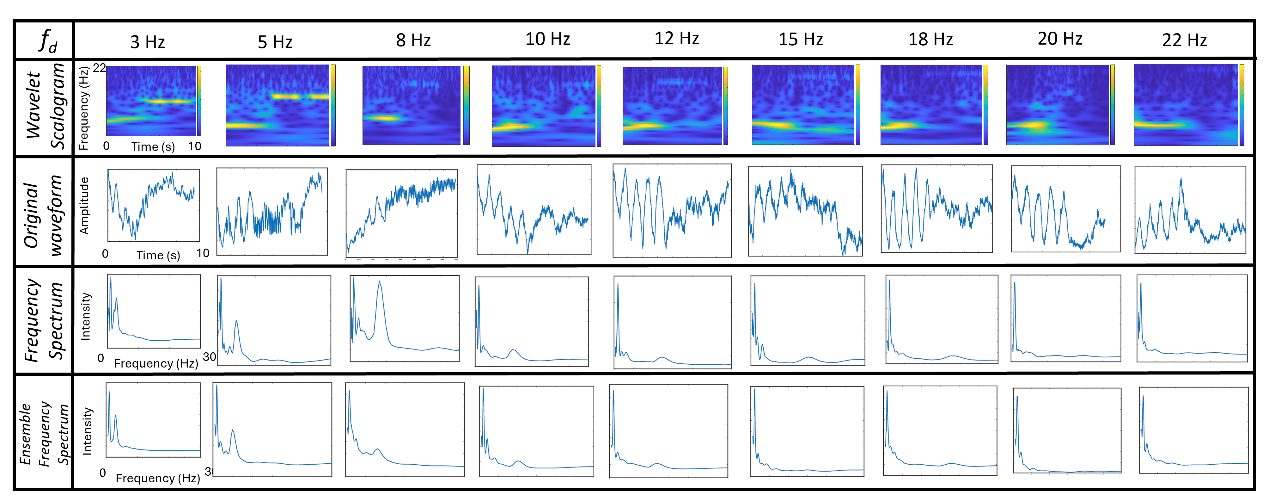
**Supplementary Figure 4.** Effects of MJP manipulation under the conditions of *V*=10 V_pp_, *H*=2.7 cm, FPS=80 Hz, *η*=0.98 cP, and *d_p_=d_mag_*=1 μm. Wavelet transform scalograms (1^st^ row), signal waveforms (2^nd^ row), frequency spectra (3^rd^ row) of representative MJPs, and ensemble frequency spectra (4^th^ row) (*n*≥90).


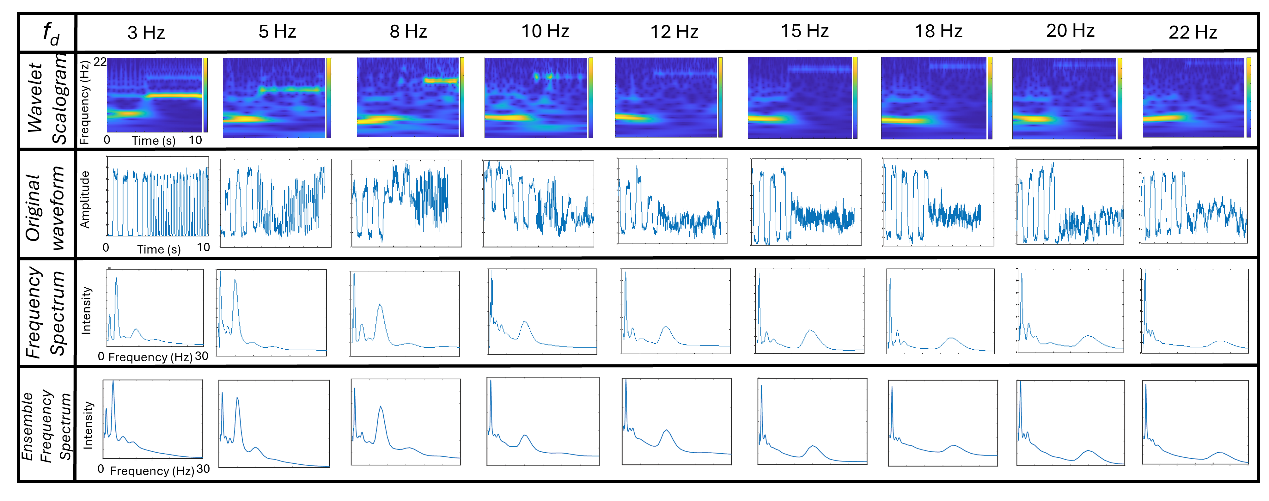


**Supplementary Figure 5.** Effects of MJP manipulation under the conditions of *V*=20 V_pp_, *H*=2.7 cm, FPS=80 Hz, *η*=0.98 cP, and *d_p_=d_mag_*=1 μm. Wavelet transform scalograms (1^st^ row), signal waveforms (2^nd^ row), frequency spectra (3^rd^ row) of representative MJPs, and ensemble frequency spectra (4^th^ row) (*n*≥90).


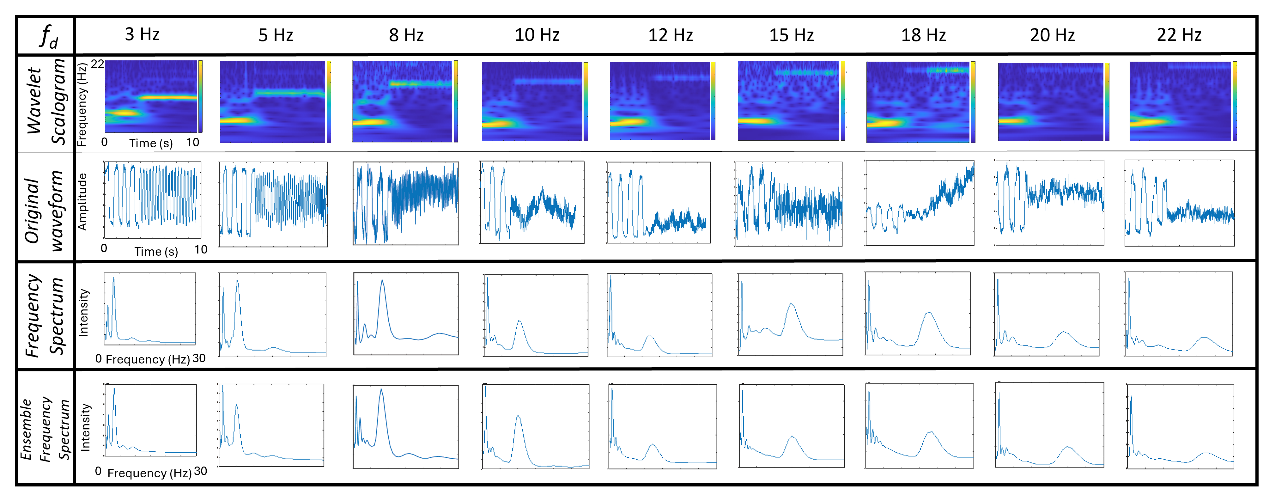


**Supplementary Figure 6.** Effect of MJP manipulation under the conditions of *V*=10 V_pp_, *H*=2.7 cm, FPS=80 Hz, *η*=0.98 cP, and *d_p_=d_mag_*=3 μm. Wavelet transform scalograms (1^st^ row), signal waveforms (2^nd^ row), frequency spectra (3^rd^ row) of representative MJPs, and ensemble frequency spectra (4^th^ row) (*n*≥25).


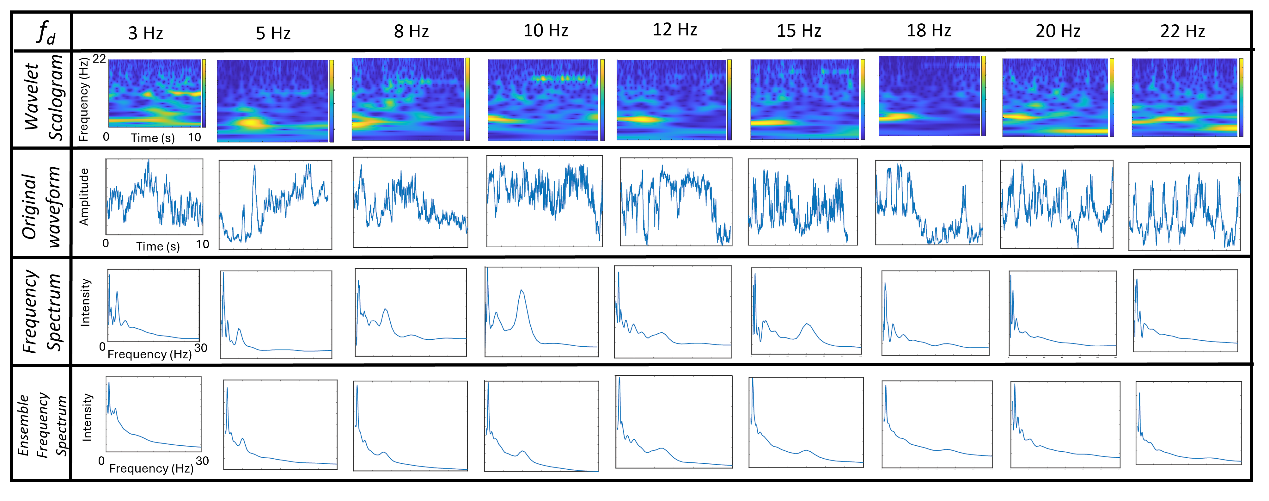


**Supplementary Figure 7.** Effects of MJP manipulation under the conditions of *V*=10 V_pp_, *H*=3.6 cm, FPS=80 Hz, *η*=0.98 cP, and *d_p_=d_mag_*=1 μm. Wavelet transform scalograms (1^st^ row), signal waveforms (2^nd^ row), frequency spectra (3^rd^ row) of representative MJPs, and ensemble frequency spectra (4^th^ row) (*n*≥90).


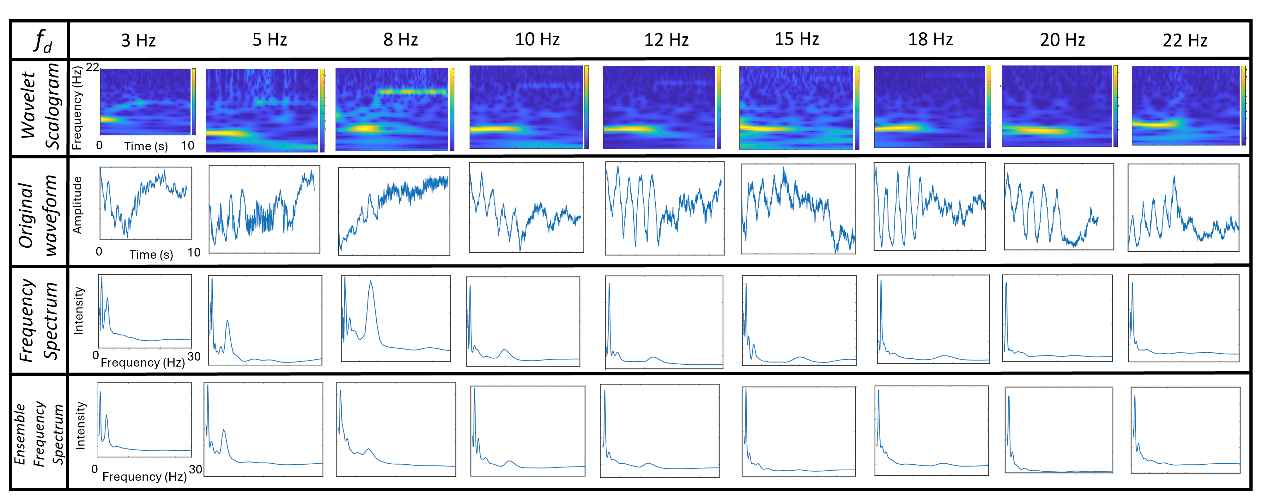


**Supplementary Figure 8.** Effects of MJP manipulation under the conditions of *V*=10 V_pp_, *H*=2.7 cm, FPS=80 Hz, *η*=7.1 cP, and *d_p_=d_mag_*=1 μm. Wavelet transform scalograms (1^st^ row), signal waveforms (2^nd^ row), frequency spectra (3^rd^ row) of representative MJPs, and ensemble frequency spectra (4^th^ row) (*n*≥90).


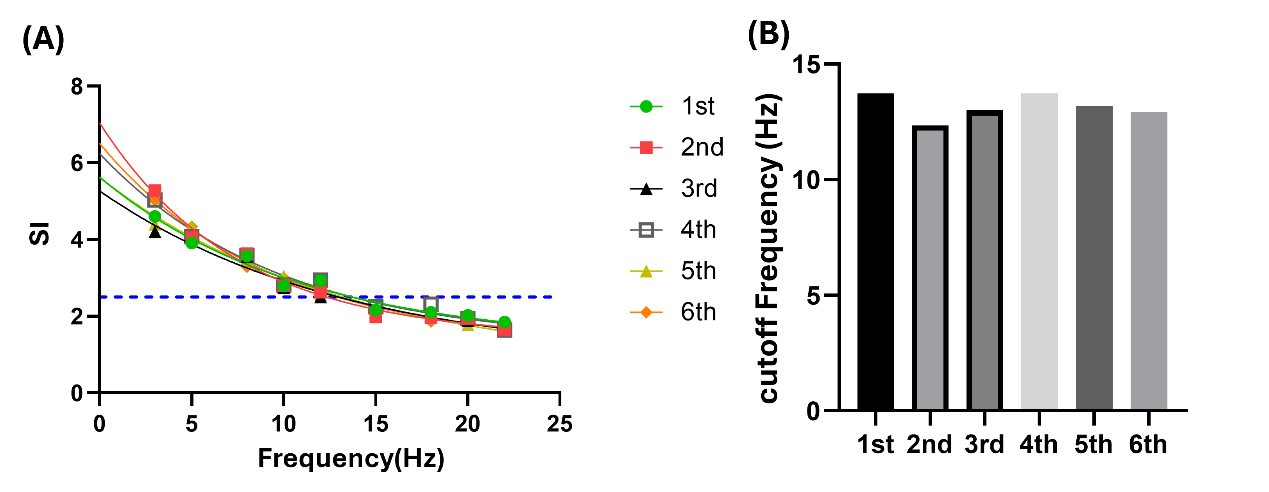


**Supplementary Figure 9.** Repeatability test. (A) Signal intensities scanned over a range of frequencies from 3 Hz to 22 Hz for 2 μm plain MJPs suspended in deionized water. The SI threshold was set as 2.5. The cutoff frequency of the six measurements is 12.9 ± 0.5 Hz, corresponding to a 3.87% variation. (B) *f_cutoff_* = 13.7, 13.2, 12.9, 13.5, 12.3, and 12.9 Hz corresponded to the first to the 6^th^ measurement, respectively.


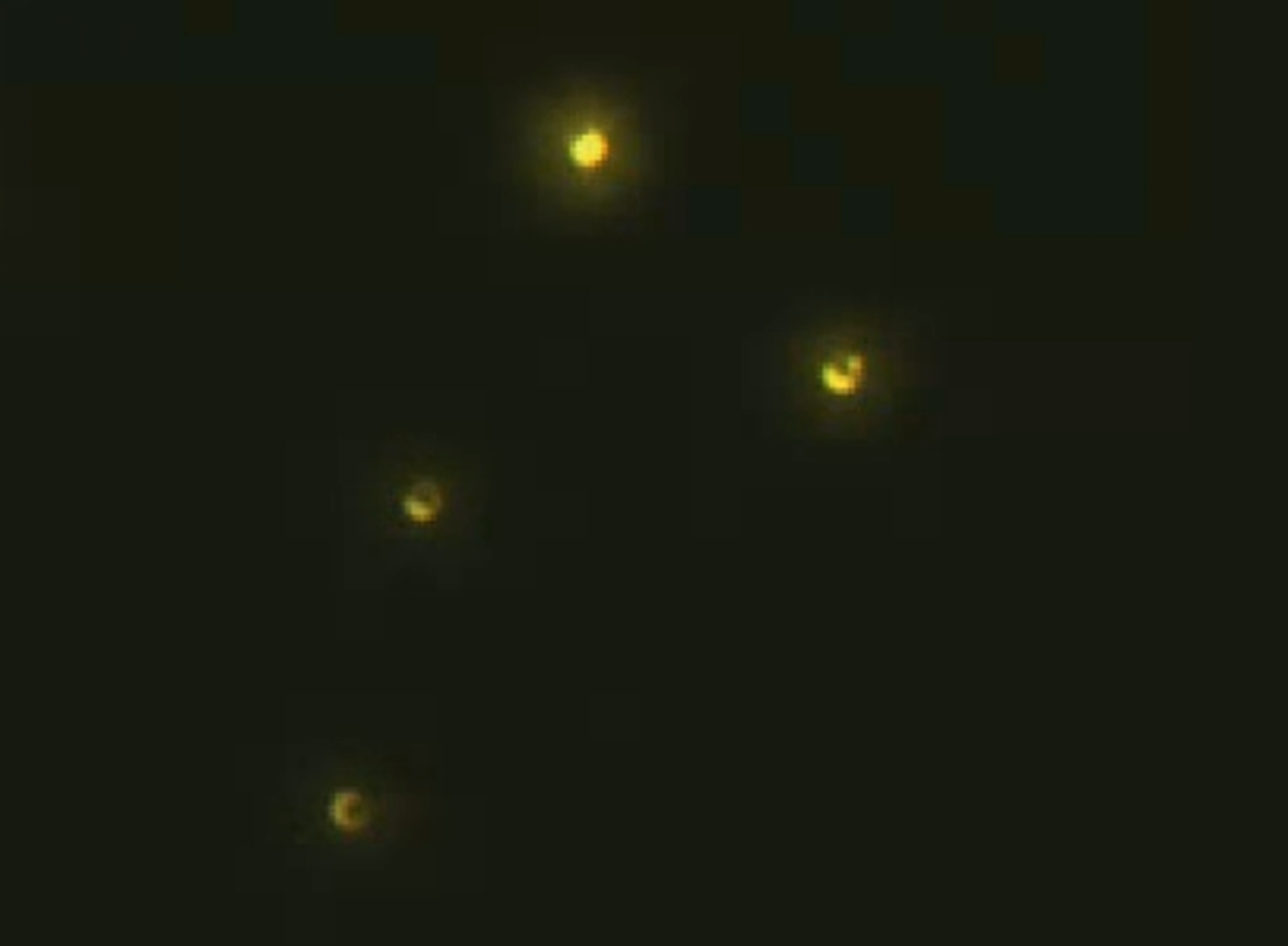


**Video S1.** Manipulation of MJPs in water captured under 20 × magnification with *d_p_* = 3 μm, *V*=10 V_pp_, *f* = 1 and 3 Hz.
